# Structural basis of βKNL2 centromeric targeting mechanism and its role in plant-specific kinetochore assembly

**DOI:** 10.1093/nar/gkag605

**Published:** 2026-06-25

**Authors:** Ramakrishna Yadala, Amanda S Camara, Surya P Yalagapati, Jitka Vaculíková, Barbora Králová, Pascal Jaroschinsky, Tobias Meitzel, Mariko Ariyoshi, Tatsuo Fukagawa, David Potesil, Twan Rutten, Jan J Palecek, Thu-Giang T Bui, Dmitri Demidov, Inna Lermontova

**Affiliations:** Leibniz Institute of Plant Genetics and Crop Plant Research (IPK) Gatersleben, Corrensstrasse 3, Seeland D-06466, Germany; Leibniz Institute of Plant Genetics and Crop Plant Research (IPK) Gatersleben, Corrensstrasse 3, Seeland D-06466, Germany; Leibniz Institute of Plant Genetics and Crop Plant Research (IPK) Gatersleben, Corrensstrasse 3, Seeland D-06466, Germany; National Centre for Biomolecular Research, Faculty of Science, Masaryk University, Kamenice 5, 62500 Brno, Czech Republic; National Centre for Biomolecular Research, Faculty of Science, Masaryk University, Kamenice 5, 62500 Brno, Czech Republic; Leibniz Institute of Plant Genetics and Crop Plant Research (IPK) Gatersleben, Corrensstrasse 3, Seeland D-06466, Germany; Leibniz Institute of Plant Genetics and Crop Plant Research (IPK) Gatersleben, Corrensstrasse 3, Seeland D-06466, Germany; UMR 1332 Biologie du Fruit et Pathologie, Univ. Bordeaux, INRAE, Villenave d’Ornon 33883, France; Graduate School of Frontier Biosciences, The University of Osaka, Suita, Osaka 565-0871, Japan; Graduate School of Frontier Biosciences, The University of Osaka, Suita, Osaka 565-0871, Japan; Central European Institute of Technology (CEITEC), Masaryk University, Kamenice 5, Brno 62500, Czech Republic; Leibniz Institute of Plant Genetics and Crop Plant Research (IPK) Gatersleben, Corrensstrasse 3, Seeland D-06466, Germany; National Centre for Biomolecular Research, Faculty of Science, Masaryk University, Kamenice 5, 62500 Brno, Czech Republic; Central European Institute of Technology (CEITEC), Masaryk University, Kamenice 5, Brno 62500, Czech Republic; Leibniz Institute of Plant Genetics and Crop Plant Research (IPK) Gatersleben, Corrensstrasse 3, Seeland D-06466, Germany; Leibniz Institute of Plant Genetics and Crop Plant Research (IPK) Gatersleben, Corrensstrasse 3, Seeland D-06466, Germany; Leibniz Institute of Plant Genetics and Crop Plant Research (IPK) Gatersleben, Corrensstrasse 3, Seeland D-06466, Germany

## Abstract

The kinetochore is an essential protein complex that ensures proper chromosome segregation during cell division. Kinetochore assembly is initiated by the incorporation of centromere-specific Histone H3 (CENP-A/CENH3) into centromeric nucleosomes. This process depends on KNL2/M18BP1 and CENP-C proteins. In eudicots, two variants of KNL2 are present, namely αKNL2 and βKNL2. Both possess the conserved SANTA domain, while αKNL2 additionally has the centromere-targeting CENPC-k motif. Despite lacking the CENPC-like motif, the plant-specific βKNL2 localizes to centromeres and aids in CENP-A/CENH3 loading. We found that efficient centromeric targeting of βKNL2 requires the SANTA domain and the *C*-terminal part, while nuclear localization is regulated by a conserved *C*-terminal motif-III, which undergoes SUMOylation. Independent experiments supported by structural analysis suggest that βKNL2 can interact multivalently with αKNL2, with DNA, and itself. We show that the centromeric targeting of βKNL2 depends on αKNL2 in a tissue-dependent manner. Our findings provide crucial insights into the unique mechanisms of plant-specific kinetochore assembly, highlighting βKNL2’s essential role in this process.

## Graphical Abstract

**Graphical Abstract:**
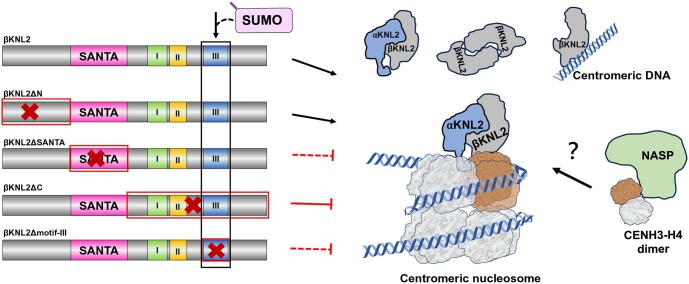

## Introduction

Kinetochores are large protein complexes that assemble on the centromere of each chromosome and connect the chromatids to the mitotic spindle. The kinetochore consists of an inner kinetochore module that is directly attached to centromeric chromatin and an outer module that interacts with spindle microtubules [1–5]. Loss of kinetochore function causes chromosome mis-segregation, aneuploidy, and cell death [6–8]. Centromere-specific Histone H3 variant (CENP-A/CENH3) nucleosomes trigger the assembly of a functional kinetochore [9], while CENP-A/CENH3 loading to centromeres involves initiation, deposition, and maintenance stages [10].

KINETOCHORE NULL2 (KNL2, also termed M18BP1) plays1 a key role in the new CENP-A/CENH3 deposition [11–13]. In vertebrates, M18BP1/KNL2 is part of the Mis18 complex, which is essential for assembling and maintaining centromeres and kinetochores during cell division [11, 14, 15]. The Mis18 complex forms an oligomeric structure with multiple copies of its subunits (Mis18α, Mis18β, and M18BP1/KNL2) [16, 17]. Cyclin-dependent kinase 1 (CDK1) regulates recruitment of the Mis18 complex to centromeres by controlling M18BP1/KNL2 oligomerization [18]. In plants, Mis18α and Mis18β have not yet been identified. In chicken and *Xenopus*, the M18BP1/KNL2 protein is present at centromeres throughout the cell cycle [19, 20] and in plants as well, except for metaphase to mid-anaphase [13].

**Figure 1.**
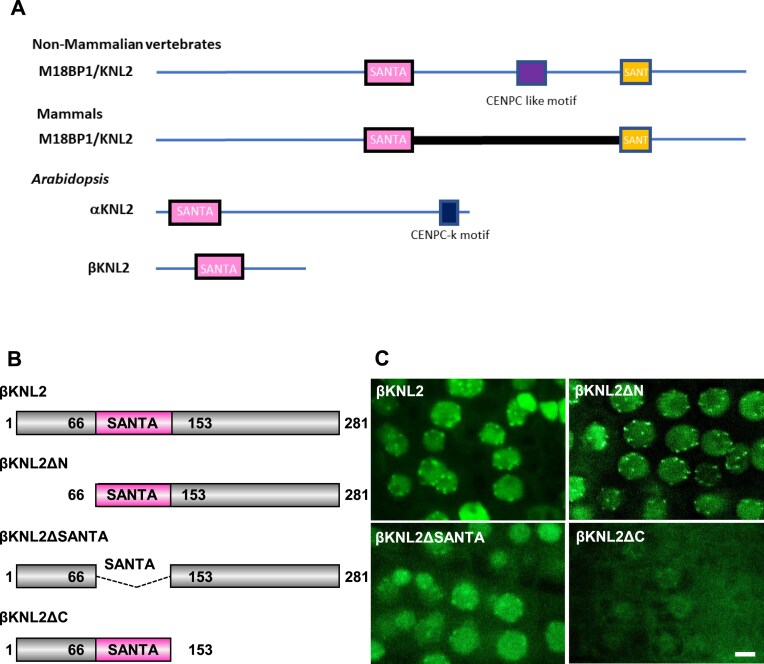
SANTA domain and the *C*-terminus of βKNL2 regulate its centromeric targeting. (**A**) Domain organization of M18BP1/KNL2 in different organisms. M18BP1/KNL2 in non-mammalian vertebrates includes SANTA (magenta) and SANT (yellow) domains and a CENPC-like motif (purple), while in mammals it includes SANTA and SANT domains separated by the CENP-C binding region (thick black line). *Arabidopsis* αKNL2 contains the SANTA domain and CENPC-k motif (blue), while βKNL2 is characterized solely by the SANTA domain. (**B**) Schemata of βKNL2 truncated variants created by site-directed mutagenesis: βKNL2ΔN lacks the *N*-terminus; βKNL2ΔSANTA lacks the SANTA domain; βKNL2ΔC lacks the *C*-terminus. (**C**) Localization patterns of EYFP-tagged βKNL2 truncated variants (referred to as 1B) in root tip nuclei of 7-day-old *A. thaliana* seedlings. The deletion of the SANTA domain and *C*-terminus affects the centromeric localization, underscoring their critical roles in targeting βKNL2 to the centromere. Scale bar 5 µm.

All identified M18BP1/KNL2 proteins typically feature the SANT-associated domain (SANTA), which is about 90 amino acids (AA) long [21, 22]. This domain is often accompanied by a SANT domain and/or a CENPC-like motif (Fig. 1A). The functional significance of the SANTA remains under debate. Deletion of the SANTA-containing part in *Arabidopsis* αKNL2 did not impair its centromere targeting [13] nor disrupted its interaction with DNA [23]. The SANTA of *Xenopus* M18BP1/KNL2 mediates its interaction with CENP-C during metaphase, thus regulating its centromere targeting [24]. However, M18BP1/KNL2 interaction with CENP-A/CENH3 nucleosomes in *Xenopus* and chicken does not require the SANTA [20, 25]. Conversely, modification of the CENPC-like motif in *Arabidopsis*, chicken, and *Xenopus* led to mis-localization of KNL2 [19, 20, 23]. In contrast to this, plant-specific βKNL2 localizes to centromeres and functions as a CENP-A/CENH3 assembly factor despite lacking a CENPC-like motif [26]. The underlying mechanisms of βKNL2 centromere targeting remain unclear and hinder our understanding of centromere and kinetochore assembly.

Unlike *αknl2* mutants, *βknl2* exhibits an embryo-lethal phenotype in *Arabidopsis* [13, 26]. We hypothesize that βKNL2 has a crucial role in centromere and kinetochore assembly. To fill the knowledge gap in plant kinetochore assembly, the present study focuses on four open questions: i) How is βKNL2 targeted to centromeres despite lacking a CENPC-like motif: which domains/motifs regulate centromeric targeting of βKNL2; ii) What promotes βKNL2’s nuclear/centromeric localization; iii) What is the position of plant-specific KNL2 in the kinetochore (inner or outer kinetochore) structure and iv) Does centromeric targeting of βKNL2 depend on other proteins? To address these questions, we combined domain mapping and targeted mutagenesis with AlphaFold3 structure prediction, identifying the motifs required for centromere targeting and for binding to kinetochore partners.

## Materials and methods

### Plasmid construction, plant transformation, and plant growth conditions

To study the localization of truncated βKNL2 variants (βKNL2ΔSANTA, βKNL2ΔN, βKNL2ΔC, βKNL2(C), βKNL2Δmotif-I, βKNL2Δmotif-II, βKNL2Δmotif-III, βKNL2Δmotif-III-SUMOprox), site-directed mutagenesis was performed on the βKNL2 cDNA clone in pDONR221 vector [26] using the Phusion site-directed mutagenesis kit (Thermo Fisher Scientific) according to the manufacturer’s protocol. All primers used are listed in Supplementary Table 1. The resulting truncated fragments in the Entry plasmid were subsequently subcloned into the Gateway-compatible expression vector pGWB641 via LR reaction (Invitrogen).

To complement the *βknl2* mutant, a genomic βKNL2 fragment including the endogenous promoter region (–756 up to + 843 relative to the transcriptional start site) was amplified from Col-0 genomic DNA using primers containing Gateway recombination sites (Supplementary Table 1) and cloned into the pDONR221 vector via a BP reaction, followed by subcloning into the expression vector pGWB640 containing the EYFP through an LR reaction.

For the *in-vitro* SUMO assay, the βKNL2 and βKNL2Δmotif-III (pDONOR221) constructs were used as templates to clone into the pET-Duet-FLAG (CM132) expression vector, which includes an *N*-terminal His-tag and a *C*-terminal FLAG-tag [27]. Individual fragments were amplified by the specific primers (Supplementary Table 1), and the PCR products were cloned into the *BamH*I site of the pET-Duet-FLAG vector using the NEBuilder HiFi DNA Assembly Kit (New England BioLabs, USA).

The cDNA from *Arabidopsis thaliana* served as a template for cloning NSE2 into the pET28c + expression vector. The PCR product was amplified using specific primers (Supplementary Table 1) and subsequently cloned into the *BamH*I and *Xho*I sites of the pET28c + vector, which carries an *N*-terminal His-tag and T7-tag.

For protein-protein interaction (PPI) studies, fragments of βKNL2 [26], βKNL2ΔSANTA, αKNL2(C), and CENP-A/CENH3 were subcloned into 3′Ven-N-pBAR GW and 3′Ven-C-pBAR GW vectors by LR reaction. These GATEWAY-compatible BiFC vectors were generated based on the highly efficient and small binary vector pPZP200BAR [28]. To this end, the cauliflower mosaic virus 35S promoter (35S) was assembled with the terminator sequence of the octopine synthase gene (tocs) from *Agrobacterium tumefaciens* via overlap extension PCR. The overlap primers were designed to create a 5´-*EcoR*I-*Spe*I-*Xba*I-3´ multiple cloning site (MCS) between the promoter and terminator sequences, and the resulting 35S:MCS:tocs fusion product was additionally flanked with restriction sites for *BamH*I and *Pst*I,

After restriction enzyme digestion using *BamH*I/*Pst*I, the 35S:MCS:tocs cassette was ligated into the linearized pPZP200BAR binary vector. To facilitate PPI analyses with the Venus fluorescent protein in all possible orientations [29], the coding sequences of both the VN (residues 1–173) and VC (residues 156–239) of Venus were amplified by PCR using primers suitable to create *EcoR*I and *Spe*I restriction sites for *N*-terminal fusions or *Spe*I and *Xba*I restriction sites for *C*-terminal fusions.

Additionally, a c-myc epitope tag was attached to both Ven_N_ variants and a hemagglutinin (HA) epitope tag to all Ven_C_ variants. The PCR fragments of the truncated Venus variants were cloned into the previously assembled binary 35S:MCS:ocs-pPZP200BAR vector using the aforementioned restriction sites. To modify the resulting BiFC binary vectors into GATEWAY-compatible destination vectors, a *Spe*I flanked PCR-fragment containing a GATEWAY *ccdB* gene cassette for LR was ligated into the linearized plasmids through digestion with *Spe*I. The final set of GATEWAY-compatible BiFC destination vectors, including 3′Ven-N-pBAR GW and 3′Ven-C-pBAR GW, was verified by sequencing. A schematic map of the GATEWAY-compatible BiFC vectors is given in Supplementary Fig. S1

In the case of the wheat germ protein expression system, βKNL2 was amplified with primers including a FLAG tag (Supplementary Table 1) from the βKNL2 cDNA cloned in pDONR221 [26]. The amplified product was then cloned into the pF3A WG (Promega) vector through restriction cloning using *Pme*I and *Sgf*I restriction enzymes. The clones were verified through colony PCR using gene-specific primers and further confirmed by Sanger sequencing.

For yeast two-hybrid (Y2H) analysis, full-length AtβKNL2 and AtαKNL2 coding sequences were subcloned from pDONR221 entry clones into the pGBKT7 and pGADT7 vectors. These entry clones were also used as templates to generate truncated fragments (αKNL2(1-118), βKNL2(50-154), and βKNL2(50-218)), which were amplified using gene-specific primers listed in Supplementary Table 1. The resulting PCR products were cloned into the *NdeI* and *BamHI* restriction sites of the pGBKT7 or pGADT7 vectors using the NEBuilder HiFi DNA Assembly Kit (New England Biolabs, USA).

*Agrobacterium*-mediated transient expression in tobacco plants was conducted following the protocol outlined by Yadala *et al*. [30]. Stable transformants of *Arabidopsis* wild-type (Columbia-0) and the *αknl2* (SALK-039432) and *βknl2* (SALK-091054) T-DNA insertion mutant backgrounds were generated using the floral dip transformation method [31]. Transgenic seedlings of all backgrounds were selected on Murashige and Skoog (MS) medium containing 20 mg/L of phosphinothricin under long-day conditions (16 h light/8 h dark). Positive transformants were further verified by PCR-based genotyping using construct-specific attB primers. In the case of transforming a heterozygous *βknl2* mutant, primary transformants were expected to include individuals with homozygous and heterozygous *βknl2* backgrounds, as well as wild-type alleles. To determine the presence and zygosity of the T-DNA insertion in the *βKNL2* gene, a combination of a T-DNA left border (LB) primer and a gene-specific right primer (SALK-091054-RP) was used, as described in Zuo *et al*. [26]. For downstream analyses, homozygous *βknl2* mutant plants expressing the genomic βKNL2 fragment fused to EYFP were selected.

*Arabidopsis* plants were initially germinated under short-day conditions (8 h light/16 h dark) and, two weeks after transplanting, grown under long-day conditions (16 h light/8 h dark).

### Protein purification and *in vitro* SUMO assay

Plasmid constructs encoding the SUMO machinery enzymes, including His-tagged SUMO1 (AT4G26840), the untagged SUMO-conjugating enzyme SCE1 (AT3G57870), and the SUMO-activating enzyme (SAE) composed of the His-tagged SAE1b subunit (AT5G50680) and the larger SAE2 subunit (AT2G21470), were obtained from Prof. Andreas Bachmair’s laboratory [32].

*Arabidopsis* SUMOylation enzymes were expressed in *Escherichia coli* BL21(DE3) RIL cells. Transformed cells were grown in LB medium at 37°C until they reached an optical density of 0.5 (OD_600_). Protein expression was induced with 1 mM IPTG at 37°C for 3 h. Cells were then pelleted and resuspended in lysis/binding buffer (50 mM phosphate buffer, 300 mM NaCl, 10 mM imidazole, 10% glycerol, 0.5% Triton X-100, pH 8.0) before sonication.

The lysate was cleared by centrifugation, and the supernatant was incubated with anti-His tag TALON affinity resin (Clontech, USA) for 1.5 h at 4°C. The resin was applied to a gravity-flow column, washed with wash buffer (50 mM phosphate buffer, 300 mM NaCl, 20 mM imidazole, pH 8.0), and eluted using buffer containing 250 mM imidazole. Elution fractions were analyzed by SDS-PAGE and Coomassie staining. Fractions containing the target protein were pooled and concentrated using Amicon Ultra Centrifugal Filter Units (10 kDa MWCO; Merck Millipore, USA). The concentrated fractions were aliquoted, and protein concentration was determined by SDS-PAGE with a BSA standard.

The βKNL2 proteins (βKNL2 and βKNL2Δmotif-III) were expressed in *Escherichia coli* BL21(DE3) RIL cells, which were grown in LB medium at 37°C until OD_600_ reached 0.5. Expression was induced by 0.5 mM IPTG at 30°C for 2 h. Cells were pelleted and resuspended in lysis/binding buffer with lower pH and 0.5 mM TCEP (50 mM phosphate buffer, 300 mM NaCl, 10 mM imidazole, 10% glycerol, 0.5% Triton X-100, 0.5 mM TCEP, pH 6.0). The remaining purification steps were identical to those described above.

The NSE2 SUMO E3-ligase was expressed under the same conditions, except induction lasted 3 h instead of 2 h. The lysis/binding buffer was used at pH 8.0. Purification followed the same procedure, with elution in 350 mM imidazole for improved yield.

The untagged SCE1 enzyme was expressed and purified in a similar way. Expression was induced by 1mM IPTG at 20°C for 1 h. After the centrifugation (4500 g, 4°C, 20 min), the pellet was resuspended in SUMO buffer (20 mM Tris-HCl, 5 mM MgCl_2_, 0.5 mM TCEP, pH 7.4). Lysate was sonicated, and the concentration of protein was determined using SDS-PAGE with the BSA standard.

For the SUMO *in vitro* assay, a similar protocol to that reported in Tomanov *et al*. [32] was used. The SUMOylation reaction mixture contained 2 μM SAE, 1.75 μM SCE1, 14 μM SUMO1, 2 μM βKNL2 protein, 7 μM NSE2 SUMO-E3 ligase, 1 × SUMO buffer, and 5 mM ATP. The reaction volume was adjusted to 20 μl with water. The mixture was incubated at 30°C for 2 h. After incubation, 20 μl of 2x Laemmli sample buffer was added, and samples were heated at 95°C for 5 min. Samples (20 μl) were separated on 12% SDS-PAGE gels, transferred to nitrocellulose membranes, reversibly stained with Ponceau S red, and analysed by immunoblotting with an anti-FLAG HRP-conjugated antibody (Abcam A8592, 1:3000).

### On-beads digestion

Following IP washes, bead-bound protein complexes were reduced using 25 mM dithiothreitol (DTT) (30 min at 56°C) and alkylated using 100mM iodoacetamide (20 min incubation at laboratory temperature in dark) with alkylation quenching done using 75mM DTT (20 min at laboratory temperature) and digested directly on beads in 50 mM NaHCO_3_ buffer using LysC (0.2 µg, Promega) for 2h at 37°C. Beads were removed from the initial digest, and the second digestion step took place using trypsin for 18 h at 37°C (0.5 µg; sequencing grade, Promega). Resulting peptides were extracted into LC-MS vials by 2.5% formic acid (FA) in 50% acetonitrile (ACN) and 100% ACN with the addition of n-Dodecyl β-D-maltoside (DDM, final concentration 0.1%; Sigma–Aldrich) and concentrated in a SpeedVac concentrator (Thermo Fisher Scientific).

### LC-MS analysis of peptides

LC-MS/MS analyses of peptide solutions were done using the UltiMate 3000 RSLCnano system (Thermo Fisher Scientific) connected to the timsTOF Ultra 2 spectrometer (Bruker). Prior to LC separation, tryptic digests were online concentrated and desalted using a trapping column (Acclaim™ PepMap™ 100 C18, dimensions 300 μm ID, 5 mm long, 5 μm particles, Thermo Fisher Scientific). After washing the trapping column with 0.1% trifluoroacetic acid, the peptides were eluted (flow rate: 150 nl/min) from the trapping column onto an analytical column (Aurora C18, 75 μm ID, 250 mm long, 1.7 μm particles, heated to 50°C, PN AUR3-25075C18-CSI, Ion Opticks) by 60 min linear gradient program (3–42% of mobile phase B; mobile phase A: 0.1% FA in water; mobile phase B: 0.1% FA in 80% ACN). Equilibration of the trapping column and the analytical column was done prior to sample injection into the sample loop. The analytical column was placed inside the Column Toaster heater (Bruker), and its emitter side was installed inside the CaptiveSpray ion source (Bruker) according to the manufacturer’s instructions, with the column temperature set to 50°C, and a spray voltage of 1.4 kV was used. PASEF data denoising was switched off.

MS data were acquired in the m/z range of 100–1700 and 1/k0 range of 0.6–1.4 V × s × cm^−2^ using the DDA-PASEF method, acquiring 10 PASEF scans with a scheduled target intensity of 20 000, and an intensity threshold of 500, and with measuring and switching times set to 2.75 and 1.65 ms, respectively. Active exclusion was set for 0.4 min with precursor reconsideration for 4 × more intense precursors. The mass spectrometry proteomics data have been deposited to the ProteomeXchange Consortium via the PRIDE (doi: 10.1093/nar/gkae1011) partner repository with the dataset identifier PXD067361.

Raw MS data were processed using Data Analysis software (version 6.1) from which MS2 spectra were exported into the MGF spectra format. MS2 spectra in MGF format were database searched using Proteome Discoverer (version 1.4, Thermo Fisher Scientific) and in-house Mascot server (version 2.6.2) in the following two-step search. At first, the data were searched against a modified cRAP database based on https://www.thegpm.org/crap/ using the following search parameters: precursor and fragment tolerance of 10 ppm and 0.03 Da, respectively, full tryptic/P specificity with 2 allowed missed cleavage sites, Oxidation (M), Deamidated (NQ), and Acetyl (Protein N-term) set as variable and Carbamidomethyl (C) as fixed modification. The peptide cutoff score for the cRAP database search was set to 30. Following the cRAP database search, the MS2 spectra assigned to any protein in the cRAP database with Mascot ion score 30 and above were removed, and the remaining spectra were searched against a specific database containing proteins of interest. Search conditions were set identically to the cRAP database search with the following modifications: additional variable modification QTGG (K), maximum number of missed cleavages 5, peptide cut-off score 20. Database search results were inspected manually in Proteome Discoverer and Data Analysis (extracted ion chromatograms), considering all acquired data inspection and especially the following characteristics: fragments assignment quality, reported fragments delta mass, Mascot ion score, and extracted ion chromatograms of the identified QTGG (K) modified peptides.

### Electrophoretic mobility shift assay (EMSA)

FLAG-βKNL2 was expressed using the TnT SP6 High-Yield Wheat Germ Protein Expression System following the manufacturer’s instructions (https://www.licor.com/bio/reagents/odyssey-emsa-kit). The expression reaction was incubated at 25°C for 2 h. The expressed protein was confirmed by Western blot analysis using anti-FLAG-tag antibodies from Sigma. The centromeric repeat *pAL1* was amplified from *Arabidopsis* Col-0 genomic DNA using IRD700-labeled and unlabeled oligos, serving as the probe and competitor, respectively. Amplicons were purified using the oligonucleotide purification kit from BioRad.

The binding reaction was set up using the Odyssey EMSA kit (LICOR) with a protocol adapted from Eysholdt-Derzsó *et al*. [33]. Motif-III extended peptide from Brassica species (Lifetain) was included in the reaction for peptide DNA binding ability testing. The expressed FLAG-βKNL2 and motif-III extended peptides with the *pAL1* labeled probe were incubated for 30 min at room temperature. The reaction was then loaded onto a 5% native polyacrylamide gel together with 6 × orange loading dye (30% glycerol, 0.25% Orange G, 10 mM Tris–HCl, pH 7.5, 1 mM EDTA) and run at 4°C with a voltage of 70V until the dye reached the bottom. Gel images were captured using a LICOR Odyssey scanner.

### Bimolecular fluorescence complementation (BiFC)

The BiFC assay was conducted following Yadala *et al*. [30]. Overnight cultures of *Agrobacterium tumefaciens* strain GV3101 carrying the desired constructs were harvested and reconstituted in agroinfiltration medium (10 mM MES, 10 mM MgCl_2_). The cultures were adjusted to 0.8 OD, and cultures expressing the testing constructs were combined in equal proportions, followed by incubation for 1 h at room temperature.

Transient expression was performed by infiltrating *Agrobacterium* culture into the abaxial side of 2–3-week-old *N. benthamiana* leaves using a 1 ml syringe without a needle. In the case of αKNL2 or αKNL2(N) interactions, leaves of *N. benthamiana* plants were infiltrated with 100 µM proteasome inhibitor (MG115 (MedchemExpress) or Bortezomib (Selleckchem)) in agroinfiltration medium 1 day after infiltration with BiFC constructs.

Fluorescence was observed 48 h post-infiltration under a Zeiss Observer fluorescence microscope (Carl Zeiss Jena GmbH, Germany), utilizing 485 nm LED excitation in combination with a 505–530 bandpass for emission detection.

### Co-immuno precipitation (Co-IP)

To perform a Co-IP experiment, βKNL2 was fused to a MYC tag and other binding partners to an HA tag. Leaves from 2–3-week-old *N. benthamiana* plants were co-infiltrated with *Agrobacterium* carrying the interaction partner plasmid on their abaxial side, following the BiFC assay infiltration protocol. As mentioned before, in the case of αKNL2 or αKNL2(N) interactions, plants were injected with 100 µM proteasome inhibitor, 1 day after agroinfiltration with the corresponding constructs. After 48 h of agroinfiltration, 2–4 grams of infiltrated leaves were harvested, and total protein extraction was performed using Phosphate buffer (50 mM NaH2PO4, 1mM β-Mercaptoethanol, 100 mM NaCl, 0.5% Nonidet-P40, pH 7–7.5). Co-immunoprecipitation (Co-IP) was conducted using the Anti-HA tag magnetic agarose trap kit (atma) and Anti-MYC tag agarose trap kit (ytak) from Proteintech, following the manufacturer’s protocol.

The leaf material was ground using a mortar and pestle in liquid nitrogen, then extracted in Phosphate buffer. After a 30-min incubation on ice, centrifugation at 4°C and 13 000 rpm (16 200 g) for 10 min, the supernatant was diluted at a ratio of 1:3 (grams:mL) and incubated with 50 μL HA trap magnetic agarose/MYC-Trap agarose. Agarose beads were incubated for 4 h at 4°C, and proteins were eluted by boiling at 95°C for 5 min with 80 μL 2 × PLB loading buffer (112 mM Na_2_CO_3_, 4 mM EDTA, 112 mM DTT, 4% SDS, 24% Sucrose, 0.02% Bromophenol Blue). A 15 μL aliquot of each sample was electrophoretically separated and transferred to a membrane, which was then probed with anti-HA (anti-HA tag McAb, Proteintech) and anti-cMYC antibodies. An extract of not infiltrated *N. benthamiana* leaves served as a negative control and was detected using a LICOR Odyssey scanner.

### Yeast two-hybrid analysis

Protein-protein interactions were analyzed using the classical Gal4-based Y2H system, as previously described [34]. Briefly, the pGBKT7 and pGADT7 constructs were co-transformed into the *Saccharomyces cerevisiae* strain PJ69-4A, and transformants were selected on SD − Leu/−Trp plates. Interaction assays were performed using drop tests on SD − Leu/−Trp/−His plates supplemented with 3-amino-1,2,4-triazole (3-AT) at concentrations ranging from 0.1 to 30 mM. Plates were incubated at 28°C. Each construct combination was co-transformed at least three times, and at least three independent drop tests were performed.

### Microscopy analysis of fluorescent signals

Confocal fluorescence imaging was performed on a Zeiss LSM780 confocal laser scanning microscope (Carl Zeiss Jena GmbH, Germany). EYFP was visualized using a 488 nm laser line for excitation in combination with a 490–550 nm band pass for detection. Images were analyzed with the Zeiss LSM software ZEN Black edition. Fluorescence loss in photobleaching (FLIP) and fluorescence recovery after photobleaching (FRAP) were recorded using a 20x water objective (N.A. 0.8) with zoom set at 4.0 and image size 512 × 80 pixels. Recordings were made with a pixel dwell time of 1 µs and a pinhole setting equaling 2 airy units. For bleaching experiments, the laser intensity was set at 100%.

FRAP experiments started with three to five pre-scans, after which an area of interest was bleached with the number of iterations adjusted to ensure a 50–70% reduction in original fluorescence. Recovery was followed until an area of stability was reached. For FLIP experiments, a small area of interest was bleached repeatedly using 1 or 5 iterations until stability was reached.

Stable transformants of *Arabidopsis* expressing fusion constructs were germinated on agar medium, and roots of 7–10-day-old seedlings were analysed for EYFP expression using a 40x water objective (NA 1.2).

### AlphaFold3 protein predictions and analyses

We used AlphaFold3 in all structural predictions of the current work [35–37]. We predicted the structures of βKNL2, αKNL2, SUMO, and Arabidopsis centromeric nucleosome in different combinations. A complete list of the complexes we tried is in Supplementary Table 2. From the prediction of each protein alone, we selected disordered regions to be removed in the prediction of complexes with the nucleosome, unless explicitly said. This selection is shown in Supplementary Fig. S2.

We analyzed interactions between βKNL2 and others by averaging the contact maps of all models where both molecules are present, with a cutoff of 20 Å. To find the regions interacting with the DNA, we summed all pairs of atoms, one from βKNL2 and the other from the DNA, less than 10 Å apart from each other and averaged over all models where βKNL2 and DNA are present. Pictures of the structures were made with PyMOL (The PyMOL Molecular Graphics System, Version 3.0, Schrödinger, Inc.). Charge distribution surface was calculated with APBS Electrostatics method inside PyMOL and PDB2PQR for calculation of Poisson-Boltzmann electrostatics [38].

Secondary structures were analyzed with the DSSP (Define Secondary Structure of Proteins) algorithm [39], and interchain interactions were calculated as the distance of a query residue to the closest residue of another chain, measured by their alpha carbons, with a cutoff of 20 Å.

## Results

### SANTA domain and *C*-terminus are required for efficient centromeric targeting of βKNL2

In plants, contrasting to other organisms, two KNL2 protein variants (α and βKNL2) containing conserved SANTA domain have been identified (Fig. 1A) [26]. But the SANTA-containing *N*-terminus of αKNL2 is not required for centromeric targeting [13, 23] and the role of the SANTA domain in βKNL2 remains elusive. Online tools like PredictProtein, LambdaPP, and DNABIND predicted that the SANTA and *C*-terminus of βKNL2 are likely involved in both PPI and protein-nucleic acid interactions (Supplementary Fig. S3).

Thus, to determine the role of the SANTA domain and other regions of βKNL2 in centromere targeting, three different truncation variants were generated: (i) a fragment lacking the *N*-terminal part (βKNL2ΔN), (ii) a fragment lacking the SANTA (βKNL2ΔSANTA), and (iii) a fragment lacking the *C*-terminal part (βKNL2ΔC) (Fig. 1B). All fragments were fused to EYFP and transiently expressed in *N. benthamiana* leaves. βKNL2ΔN and βKNL2ΔSANTA localized to centromeres and the nucleoplasm, resembling the full-length protein used as a control, whereas βKNL2ΔC was detected mainly in the cytoplasm and nucleoplasm (Supplementary Fig. S4). To quantify these patterns, three independent plants per construct were analyzed, with 50 cells scored per plant (Supplementary Fig. S5A). Compared with the control, βKNL2ΔN and particularly βKNL2ΔSANTA showed a reduced number of cells with centromeric + nucleoplasmic signal pattern, while βKNL2ΔC was predominantly cytoplasmic + nucleoplasmic, with only a small fraction of cells exhibiting centromeric localization pattern (Supplementary Fig. S5A). We generated stable *Arabidopsis* lines expressing each EYFP-tagged construct and analyzed the localization of the corresponding fusion proteins in 10–12 T_2_ seedling root tips of three independent transgenic lines. βKNL2ΔN exhibited centromeric localization similar to that of full-length βKNL2 (Fig. 1C). However, βKNL2ΔSANTA transformants showed reduced centromere association, whereas βKNL2ΔC transformants had mis-localization to the cytoplasm and nucleoplasm, occasionally showing weak centromere-like signals similar to those observed in transient expression (Fig. 1C, Supplementary Fig. S4). This was further confirmed by immunostaining in *Arabidopsis*, where βKNL2ΔSANTA and βKNL2ΔC showed hardly detectable centromeric signals, demonstrating diffuse and non-specific nucleoplasm signals instead (Supplementary Fig. S5). Thus, efficient centromeric localization of βKNL2 likely depends on both the SANTA domain and the *C*-terminal region.

### Motif-III in the *C*-terminus of βKNL2 is required for the nuclear localization of βKNL2

**Figure 2.**
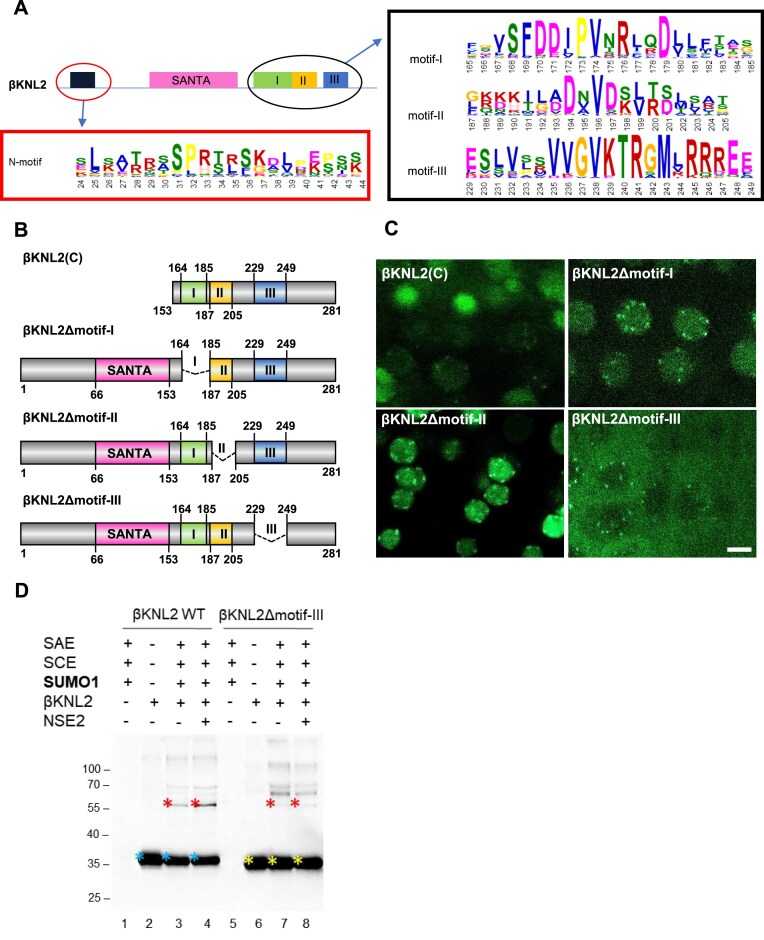
βKNL2 nuclear localization is regulated by conserved *C*-terminal motif-III. (**A**) Schematic representation of the βKNL2 protein structure. Newly identified conserved motifs at the *N*- and *C*-termini of βKNL2 are encircled and accompanied by WebLogo alignments showing conserved AA across dicots. (**B**) Truncated constructs of βKNL2 to elucidate the functional role of the entire *C*-terminus and its individual motifs. The βKNL2(**C**) variant retains only the *C*-terminal region, while the βKNL2Δmotif-I, βKNL2Δmotif-II, and βKNL2Δmotif-III variants omit one of the three corresponding motifs. (**C**) Localization patterns of EYFP-tagged βKNL2 truncated protein variants (shown in Fig. 2B) in *Arabidopsis* root tip nuclei. βKNL2(**C**) showed reduced centromeric localization. βKNL2Δmotif-I and βKNL2Δmotif-II fragments displayed normal nuclear and centromeric signals, whereas βKNL2Δmotif-III exhibited mislocalization to the cytoplasm, indicating the critical role of motif-III in nuclear targeting or retention. Scale bar 5 µm. (**D**) *In vitro* SUMOylation assay of βKNL2 variants using SUMO1. Lanes 1 and 5: enzymes only; lanes 2 and 6: substrate only; lanes 3 and 7: enzymes + substrate; lanes 4 and 8: complete reactions with NSE2 (SUMO E3 ligase). Proteins were detected with anti-FLAG antibody. Blue asterisk: unmodified βKNL2 WT; yellow asterisk: βKNL2Δmotif-III; red asterisks: SUMOylated forms. βKNL2Δmotif-III shows reduced SUMOylation, while NSE2 enhances modification only in WT.

Previously, we identified four βKNL2-specific conserved motifs [26]. Extended sequence analysis of approximately 100 KNL2 variants without the CENPC-k motif from monocots and dicots (Supplemental File.1) confirmed that the conserved motifs are specific to βKNL2. Each motif spans approximately 20 AA (Fig. 2A, Supplemental File.2). The first motif (N-motif, residues 24–44) is located at the *N*-terminus, upstream of SANTA, while the other three: motif-I (residues 165–185), motif-II (residues 187–205), and motif-III (residues 229–249), are positioned in the *C*-terminus, directly downstream of the SANTA (Fig. 2A).

To explore whether the *C*-terminus alone is capable of localizing to the centromere, we generated a truncated construct containing solely the *C*-terminal part (βKNL2(C)) fused to EYFP (Fig. 2B). This variant is primarily localized to the nucleoplasm, with only occasional weak centromeric signals observed in a small subset of nuclei in stably transformed *Arabidopsis* and transiently transformed *N. benthamiana* (Fig. 2C, Supplementary Fig. S6). This indicates that βKNL2(C) has minimal centromere association and confirms our assumption that efficient centromeric targeting of βKNL2 requires both the *C*-terminal part and the SANTA domain.

Furthermore, the influence of *C*-terminal motifs on βKNL2 localization was tested by generating constructs with individual deletions of each motif, which were then fused to EYFP (Fig. 2B). These constructs were then employed for transient expression in *N. benthamiana* and for stable expression in *A. thaliana*. In *N. benthamiana* leaves transiently expressing βKNL2Δmotif-I or βKNL2Δmotif-II, fluorescence was predominantly detected at centromeres and in the nucleoplasm, whereas the βKNL2Δmotif-III-EYFP fusion protein showed cytoplasmic + nucleoplasmic or centromeric + cytoplasmic signal patterns (Supplementary Figs. S6, S7). Consistently, T_2_ lines of *A. thaliana* expressing βKNL2Δmotif-I and βKNL2Δmotif-II constructs showed fluorescence at centromeres and in the nucleoplasm, while βKNL2Δmotif-III lines exhibited mis-localization to the cytoplasm, with clearly detectable centromeric but strongly reduced nucleoplasmic signals (Fig. 2C, Supplementary Fig. S6). Immunostaining using anti-GFP antibodies further confirmed that the EYFP signals of βKNL2Δmotif-I and βKNL2Δmotif-II constructs colocalized with chromocenters, where centromeres are organized in *Arabidopsis* (Supplementary Fig. S7). Whereas βKNL2Δmotif-III-EYFP signals are localize at chromocenters and reduced nucleoplasmic staining, suggesting impaired nuclear distribution.

BLASTP analysis of “motif-III” identified numerous matches across eukaryotes and prokaryotes, particularly with transcription factors and nucleic acid-binding proteins. Further analysis of motif-III with ELM and GPS-biocuckoo revealed a potential SUMOylation site spanning residues 221–231, which overlaps with motif-III (residues 229 to 249) (Supplementary Fig. S8, Supplemental File.3). Previous studies show that SUMOylation acts as a signal for protein nuclear import and export [40–42]. This suggests that the loss of motif-III may disrupt SUMOylation, potentially explaining the cytoplasmic mislocalization of βKNL2Δmotif-III.

### βKNL2 undergoes SUMOylation at the motif-III site

Post-translational modifications, such as SUMOylation, can play crucial roles in regulating protein interactions, function, and localization. To determine whether βKNL2 undergoes SUMOylation and whether specific motifs within its sequence contribute to this modification, we performed *in vitro* SUMOylation assays. Purified βKNL2 and βKNL2Δmotif-III were incubated at 30°C for 2 h with a minimal enzymatic system comprising the E1 SUMO-activating enzyme (SAE), E2 SUMO-conjugating enzyme (SCE), and the SUMO1 isoform. Additionally, SUMOylation efficiency was tested in the presence of the NSE2 E3 SUMO-ligase.

Western blot analysis using anti-FLAG antibodies revealed distinct higher molecular weight bands corresponding to SUMOylated forms of βKNL2 (Fig. 2D, lane 3). The intensity of these bands increased upon addition of NSE2 (lane 4), indicating that SUMOylation is enhanced by this E3 ligase. In contrast, βKNL2Δmotif-III exhibited a significant reduction in SUMOylation (Fig. 2D, lanes 7 and 8), suggesting that motif-III contains a key SUMOylation site essential for this post-translational modification. The observed decrease in SUMOylation efficiency in the mutant variant indicates that motif-III contains a lysine residue that serves as a SUMO acceptor site.

To pinpoint the specific residue, we performed mass spectrometry analysis, which identified lysine 229 within motif-III as the SUMOylation site. Although this lysine is neither conserved nor part of the canonical SUMOylation consensus motif (Ψ-K-x-E or φ-K-x-E), it lies within an acidic region enriched with aspartic acid residues, which may promote SUMO conjugation.

Further, we designed a new truncated construct (βKNL2Δmotif-III-SUMOprox) that includes the predicted SUMOylation region but excludes the conserved region of motif-III (Supplementary Fig. S6). The lines expressing βKNL2Δmotif-III-SUMOprox showed partial recovery of nucleoplasm localization compared to βKNL2Δmotif-III (Supplementary Fig. S6 and S7A).

These findings suggest that SUMOylation of βKNL2 is dependent on the presence of motif-III and is further facilitated by the NSE2 SUMO ligase. Given that SUMOylation often regulates protein stability, localization, and interactions, this modification could play an important role in βKNL2 function within the nucleus and kinetochore complex.

### βKNL2 binds to the centromeric DNA *in vitro*

**Figure 3.**
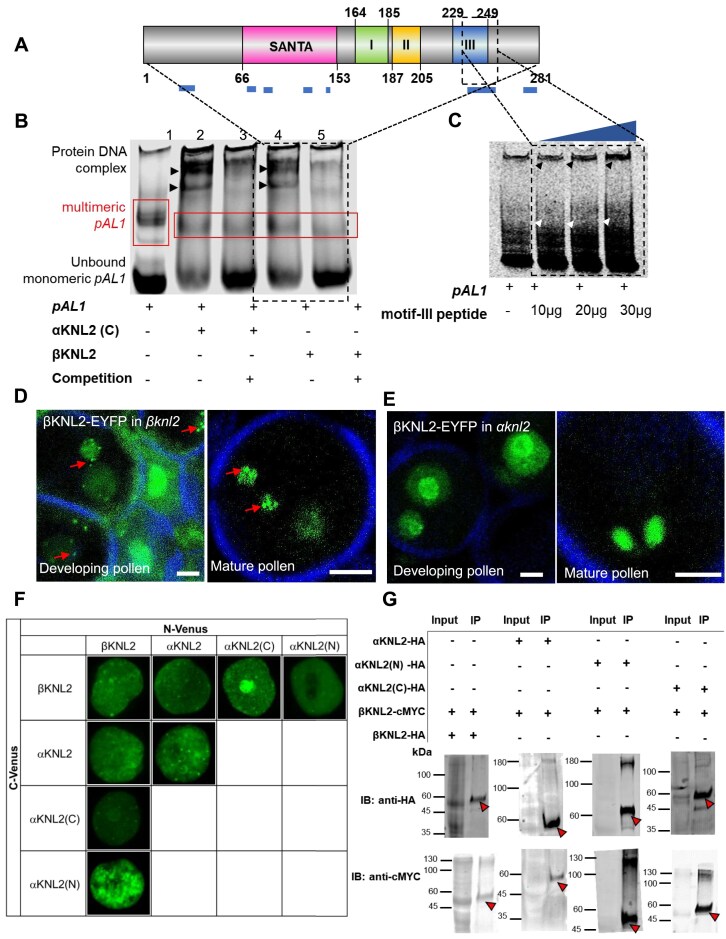
βKNL2 binds to centromeric DNA, and its centromeric recruitment depends on αKNL2 in pollen nuclei. (**A**) Schematic representation of the βKNL2 protein with predicted DNA-binding regions marked by blue bars below. (**B**) EMSA on native gel showing FLAG-βKNL2 binding to centromeric *pAL1* DNA. IRD700-labeled *pAL1* monomer and multimeric repeats probes were amplified from *Arabidopsis* genomic DNA (lane 1). IRD700-*pAL1* probes shifted upwards, indicating binding by *in vitro* expressed FLAG-αKNL2(**C**) as a positive control (lane 2) and FLAG-βKNL2 (lane 4). Competition with an excess of unlabeled *pAL1* DNA reduced the shifts, confirming specific binding of FLAG-αKNL2 (lane 3) and FLAG-βKNL2 (lane 5) to *pAL1* DNA. (**C**) Motif-III of βKNL2 can bind to centromeric DNA. Increased upward shifts correlate with increasing concentrations of motif-III peptide (4.6, 9.1, and 13.6 µM; corresponding to 10, 20, and 30 µg, respectively) in IRD700-labeled *pAL1* DNA, demonstrating dose-dependent binding. (D-E) Disturbed centromeric localization of βKNL2-EYFP in *αknl2* compared to *βknl2* mutant pollen nuclei, indicating centromeric recruitment of βKNL2 is dependent on αKNL2. (**D**) In *βknl2* mutant transformants, βKNL2-EYFP localizes to centromeres and nucleoplasm in the nuclei of developing and mature pollen. Red arrows highlight centromere-like puncta, with a maximum of five signals observed in sperm nuclei. (**E**) In *αknl2* mutant transformants, βKNL2-EYFP fluorescence is localized in the nucleoplasm and nucleoli, with no observable centromere-like signals. Pollen wall autofluorescence in blue. Scale bar: 5 µm. (**F**) BiFC assays for confirmation of βKNL2 interactions: Positive interactions were observed between βKNL2:βKNL2, βKNL2:αKNL2, βKNL2:αKNL2(**N**), and βKNL2:αKNL2(**C**). (**G**) Western blots with anti-HA and anti-cMYC tag antibodies display monomeric size bands (red arrow heads) from co-immunoprecipitation (co-IP) performed with anti-HA trap.

Different inner kinetochore proteins associate with the centromeric DNA [2, 4, 5, 25]. For example, αKNL2 associates with DNA in a sequence-independent manner *in vitro* and preferentially with *pAL1 in vivo* [23, 43]. Different predictions show that βKNL2 has the ability to interact with nucleic acids (Supplementary Fig. S3) at the regions shown in Fig. 3A. To validate this, we performed Electrophoretic Mobility Shift Assay (EMSA) using IRD700 labelled *pAL1* DNA (178 bp *A. thaliana* centromeric DNA) and *in-vitro* expressed FLAG-βKNL2. FLAG-αKNL2(C) served as a positive control, and expression of both proteins was verified by Western blot (Supplementary Fig. S9). IRD700-*pAL1* was amplified from *Arabidopsis* genomic DNA, so it contained monomeric and multimeric repeats of *pAL1* (Supplementary Fig. S9).

We observed an upward shift in the lane of IRD700-*pAL1* when incubated with FLAG-βKNL2, similar to FLAG-αKNL2(C) (Fig. 3B lanes 2 and 4). However, when excess unlabeled *pAL1* was used as a competitor along with labeled probes, a reduced shift was observed, indicating successful competition (Fig. 3B, lanes 3 and 5). The intensity of the shifted bands was further quantified using ImageJ analysis (Supplementary Fig. S10), which revealed a decrease in shift intensity in the presence of competitor DNA, supporting the interaction.

As mentioned earlier, BLAST analysis of motif-III resulted in several hits with various nucleic-acid binding proteins. To verify if motif-III is capable of binding DNA, we synthesized a peptide containing motif-III (AA238 VK/ETRGMLRRREEY/GEASIGK/ER AA257). In the EMSA assay using 30 femtomoles of *pAL1*, increasing concentrations of the peptide (4.6 µM, 9.1 µM, and 13.6 µM; corresponding to 10, 20, and 30 µg, respectively) resulted in a progressively enhanced DNA shift (Fig. 3C). The intensity of the shifted DNA-peptide complex was quantified using ImageJ, and a clear increase in shift signal was observed with increasing peptide concentrations (Supplementary Fig. S10). These results suggest that motif III of βKNL2 could bind to centromeric DNA.

### βKNL2 depends on a CENPC-like motif containing αKNL2 for centromeric localization

Previous studies have shown that the M18BP1/KNL2 requires the CENPC-like motif for centromeric localization [17, 19, 23, 25]. Interestingly, βKNL2 exhibits centromeric localization even without this motif. In our previous study, we hypothesized that βKNL2 might rely on CENPC-like motif-containing proteins (αKNL2 or CENP-C) for its centromere targeting [26].

To test this hypothesis, we introduced the βKNL2::βKNL2-EYFP construct into *αknl2* (-/-) and *βknl2* (±) mutants. Complemented plants were selected as described in the Materials and methods. We expected reduced centromeric localization of βKNL2-EYFP in *αknl2* mutants, reflecting βKNL2 dependency on αKNL2. Since both KNL2 genes are highly expressed in meristematic cells, we assessed βKNL2-EYFP localization in floral tissues of both mutants.

We initially focused on pollen (Fig. 3D, E, Supplementary Fig. S11). In *βknl2* mutants, βKNL2-EYFP was present at the centromeres and nucleoplasm of both generative and vegetative nuclei in bi-nucleate pollen, and up to five distinct centromere-like signals were observed in the sperm nuclei of mature tri-nucleate pollen (Fig. 3D). Interestingly, vegetative nuclei of mature pollen showed exclusive nucleoplasm localization of the fluorescence. In contrast, *αknl2* mutant transformants displayed βKNL2-EYFP fluorescence in both the nucleoplasm and nucleolus, but lacked distinct centromeric signals in bi-nucleate and tri-nucleate pollen nuclei. (Fig. 3E, Supplementary Fig. S11).

Next, we examined developing seeds with embryos, and both *αknl2* and *βknl2* transformants showed similar centromeric localization patterns in the embryo, although the signal appeared slightly stronger in the *βknl2* background (Supplementary Fig. S11). Notably, other tissues in the developing seeds displayed disrupted centromeric localization similar to that observed in pollen nuclei. These results suggest that the centromeric localization of βKNL2 partially depends on αKNL2 in a tissue-specific manner.

### βKNL2 interacts with itself and αKNL2

Across various organisms, kinetochore components tend to form homodimers and oligomers [18, 44, 45]. In animals, the Mis18 complex is formed by an M18BP1/KNL2 homodimer and a hetero-oligomer of Mis18α&β. Notably, plants possess two KNL2s (α&βKNL2) and lack the Mis18α&β. Thus, raises the question of whether βKNL2 can dimerize, potentially fulfilling a role analogous to the Mis18 complex.

To investigate the potential for homo- and heterodimer formation, we performed BiFC assays. βKNL2 and αKNL2 were fused to the N (NV)- and the C (CV)-terminus of Venus in both directions. To surpass overexpression difficulty of αKNL2 due to its proteasomal regulation [13], we applied the 26S proteasome inhibitor bortezomib, which has been shown to enhance αKNL2 accumulation and centromeric localization [46]. Fluorescence was observed when βKNL2-NV was co-infiltrated with βKNL2-CV, or αKNL2-CV, indicating that βKNL2 is capable of forming both homomeric and heteromeric interactions (Fig. 3F).

To map the specific interaction domains of αKNL2 with βKNL2, we performed a BiFC assay using truncated αKNL2 constructs, αKNL2(N) (residues 1–362, containing the SANTA) and αKNL2(C) (residues 363–598, with CENPC-k motif). Both αKNL2(N) and αKNL2(C) showed interaction with βKNL2 in both directions (Fig. 3F). In all combinations tested, fluorescence was consistently observed in the nucleoplasm along with punctate centromeric dot patterns. This suggests that βKNL2 and αKNL2 not only interact within the nucleoplasm but also possibly co-localize at centromeres, supporting their functional association in kinetochore assembly.

To validate the interactions of βKNL2 with αKNL2 variants, we performed a co-IP assay using an anti-HA trap (Fig. 3G). For this, βKNL2-MYC-NV was transiently co-expressed in tobacco leaves as the primary interaction partner together with one of the secondary partners: βKNL2-HA-CV, αKNL2-HA-CV, αKNL2(N)-HA-CV, or αKNL2(C)-HA-CV.

Following immunoprecipitation with an anti-HA trap, Western blot analysis using anti-MYC antibodies detected monomer-sized bands between 45 and 60 kDa in all four cases. These observed bands were higher than the predicted molecular weight of 43.42 kDa for βKNL2-HA-CV, suggesting post-translational modifications such as SUMOylation.

Together, BiFC and co-IP results support a close association between βKNL2 and αKNL2, potentially through dimerization, although the exact interaction interface remains to be validated.

### Structural models of βKNL2 suggest possible assembly with αKNL2, SUMO1, and the nucleosome

**Figure 4.**
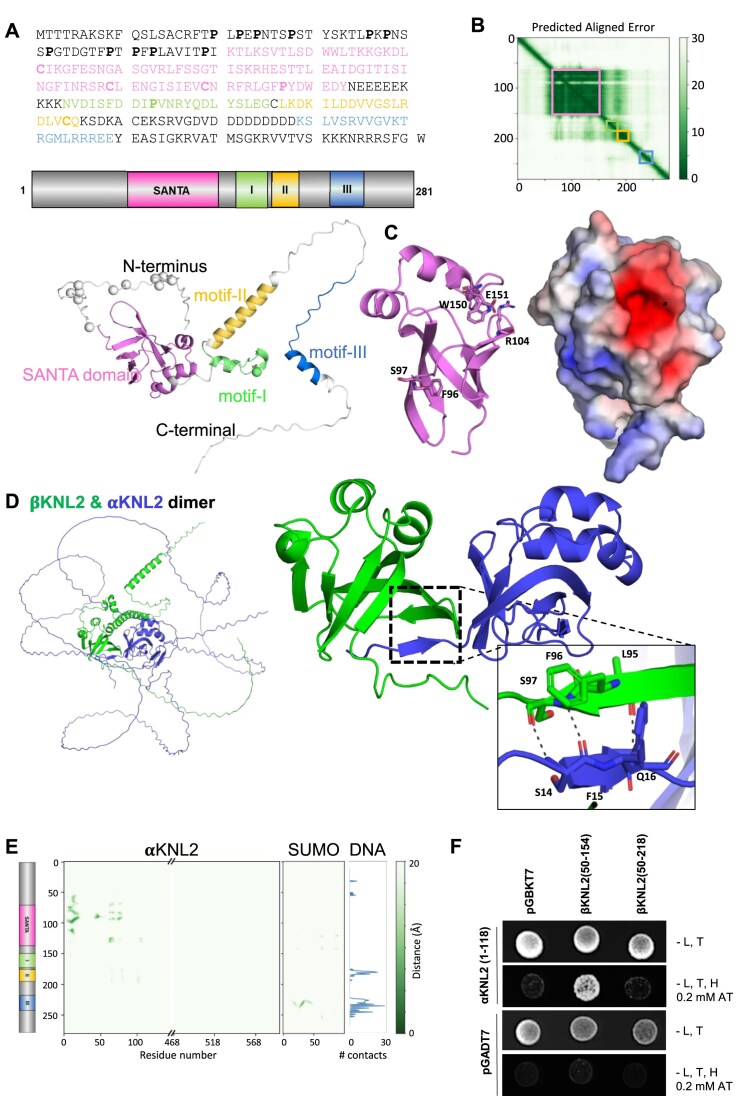
Structural prediction of βKNL2 and its complexes. (**A**) Sequence (top) and predicted structure (bottom, in cartoon mode) of βKNL2 highlighting the SANTA domain (pink) and identified motifs I (green), II (yellow), and III (blue). Prolines, enriched in the *N*-terminal, are marked in bold in the sequence and with small spheres in the structure. (**B**) Predicted Aligned Error output by AlphaFold’s prediction. The SANTA domain and the *C*-terminal motifs are identified by squares following the color code in (**A**). (**C**) The structure of the SANTA domain in cartoon mode with key residues in sticks (left) and in surface mode, colored by surface charge distribution (right), where red and blue regions are negatively and positively charged, respectively. The black asterisk marks the negatively charged pocket. (**D**) Structural prediction of the complex of βKNL2 (green) and αKNL2 (blue) entirely (left) and only their SANTA domains (right), with a zoom on the intermonomeric β-sheet showing hydrogen bonds (dashed black lines) between key residues of both monomers drawn in stick mode. (**E**) Averaged contacts between βKNL2 (*y*-axis) and αKNL2, SUMO, or DNA (*x*-axis). Contacts between protein molecules are shown as contact maps, distinguishing residues of both molecules, while contacts between βKNL2 and DNA only distinguish which AA residues contact the DNA, regardless of which DNA base pair. Averages were calculated over all structural models where both molecules are present. (**F**) Yeast two-hybrid assay showing interaction between αKNL2(1–118) and βKNL2(50–154). Upper row: growth control on medium lacking leucine and tryptophan (−Leu, −Trp). Middle row: interaction test on selective medium lacking leucine, tryptophan, and histidine (−Leu, −Trp, −His) supplemented with 0.2 mM 3-amino-1,2,4-triazole. A positive interaction was observed for Gal4AD-αKNL2(1–118) and Gal4BD-βKNL2(50–154) (middle column), whereas Gal4AD-αKNL2(1–118) and Gal4BD-βKNL2(50–218) did not interact (right column). Lower row: empty pGBKT7 and pGADT7 vectors used as negative controls on selective medium.

Experimentally solving the structure of a KNL2 protein is challenging. Two short pieces of it have been solved by Cryo-EM and NMR - the CENPC-like domain of chicken KNL2, together with a CENP-A nucleosome (PDB code 7y7i, Jiang *et al*. [25]), and a MYB-like DNA binding domain of *C. elegans* KNL2 (PDB code 2m3a), respectively. More recently, the SANTA domain of human M18BP1 has been solved by crystallography (PDB not yet released, Walstein *et al*. [47]). Nevertheless, AlphaFold3 [37] predicts the SANTA domain of βKNL2 with high confidence, and motifs I, II, and III with confidence above 50 (Fig. 4A and B).

The predicted structure of the SANTA domain of *Arabidopsis* βKNL2 (Fig. 4C) closely resembles that of human M18BP1 described by Walstein *et al*. [47]. All β-strands adopt the same fold, and several conserved AA occupy equivalent positions. For example, R104 and W150 in βKNL2 correspond to R421 and W467 in M18BP1. However, the hydrophobic cavity described in M18BP1 is very negative in βKNL2, adjacent to E151, which lies at the same position as the conserved K468 in M18BP1. When this region is compared with the *Arabidopsis* αKNL2 SANTA domain, αKNL2 displays less negative charge and a more hydrophobic character than βKNL2 (Supplementary Fig. S12). Another notable difference is the replacement of the conserved W413 and H414 in M18BP1 by F96 and S97 in βKNL2, located in β-strand β4. Walstein *et al*. [47] proposed that these residues are important for M18BP1 kinetochore localization, although they are not the sole determinants. In our structural predictions, the equivalent region in βKNL2 consistently interacts with αKNL2.

We investigated the interaction of βKNL2 with αKNL2, SUMO1, itself, and the nucleosome in different combinations using AlphaFold3 (Supplementary Table 2). In all different models where βKNL2 and αKNL2 are present, they are consistently predicted to interact through their SANTA domains (Fig. 4D, Supplementary Fig. S13). The *N*-terminal tail (pre-SANTA) of αKNL2 folds back, making hydrogen bonds with F96 and S97 of βKNL2’s SANTA domain, forming an intermonomeric β-sheet. This interaction is supported by averaged contact maps (Fig. 4E), as well as the proximity of the *C*-terminal region of βKNL2 to the αKNL2 SANTA domain. In this arrangement, the aforementioned electronegative pocket of βKNL2 remains exposed for further interactions.

To further delineate the interaction between βKNL2 and αKNL2, we performed Y2H assays using both full-length and AlphaFold-guided truncations (Fig. 4D,E). Although full-length αKNL2 and βKNL2 did not show detectable interaction, Gal4AD-αKNL2(1–118) specifically interacted with Gal4BD-βKNL2(50–154) (Fig. 4F). By contrast, Gal4BD-βKNL2(50–218), which extends beyond the SANTA domain toward motif I, did not interact. These findings indicate that the interaction between αKNL2 and βKNL2 is mediated by the SANTA-containing regions. The absence of interaction in the full-length proteins is likely due to reduced accessibility of this interface in the context of the complete protein.

In contrast, βKNL2’s interactions with itself and SUMO1 are less consistent across different structural models (Supplementary Fig. S14 and 15). The *C*-terminal region of βKNL2 emerges as a key interaction hub, exhibiting structural flexibility and multivalently engaging in diverse interactions: with SUMO1, primarily through motif III (Fig. 4E, Supplementary Fig. S14); with the *C*-terminal region of other βKNL2 molecules, as seen in models with a homodimer (Supplementary Fig. S14); and with DNA in models that include the nucleosome (Fig. 4E, Supplementary Fig. S18). Notably, we observed conformational changes in the *C*-terminal motifs when βKNL2 formed dimeric or trimeric assemblies (Supplementary Fig. S16).

Structural predictions of βKNL2 and αKNL2 in complex with the nucleosome show that the αKNL2 CENPC-k motif adopts the same position as in the structure reported by Jiang *et al*. [25]. These models consistently showed the interaction between SANTA domains through the described intermonomeric β-sheet, while exposing the electronegative pocket of βKNL2 and the SUMOylation site (except when predicted with SUMO, which binds there) (Supplementary Fig. S18). However, the location of β- and αKNL2-bound SANTA domains varied relative to the histones across models, and the DNA was often predicted to adopt a kinked conformation, complicating the identification of specific interactions between the SANTA domains and the nucleosome (Supplementary Fig. S18).

### βKNL2 is highly dynamic and loosely bound to the centromeric nucleosome

We performed FRAP experiments to track the dynamics of βKNL2-EYFP within *Arabidopsis* elongated nuclei (Supplementary Fig. S17). These nuclei are structurally stable and less active, and they do not move out of focus. Repeated FRAP showed that βKNL2-EYFP has high mobility, as indicated by rapid fluorescence reduction followed by swift recovery. This observation was supported by a single FRAP, which revealed rapid recovery of fluorescence with a short half-time (t1/2) of approximately 0.7 seconds. Similar turnover rates and mobility were also observed for EYFP-αKNL2 [13], indicating that both KNL2 proteins bind flexibly to nucleosomes.

We used AlphaFold3 to model how KNL2 proteins bind to centromeric nucleosomes (Supplementary Fig. S18). This model aligns with the experimentally resolved structures, like the αKNL2 CENPC-k motif, which binds to histones similar to the ggKNL2 CENPC-like motif [25]. They also consistently showed the interaction between SANTA domains as described above (Supplementary Fig. S18).

## Discussion

In metazoans, a single M18BP1/KNL2 variant is maintained, while plants have evolved different versions: α- and βKNL2 in eudicots, and γ- and δKNL2 in grasses. Notably, the centromeric localization of αKNL2 in eudicots depends on the presence of the CENPC-k motif [23]. In contrast, βKNL2 localizes to centromeres even without this motif [26]. Analysis of the βKNL2 sequence revealed four specific conserved motifs: one in the *N*-terminus and three in the C-terminus relative to the SANTA domain.

Our study provides insight into how the plant-specific βKNL2 contributes to centromere assembly through a combination of protein-protein interactions, DNA binding, and post-translational regulation.

Our experimental data show that while deletion of the *N*-terminus of βKNL2 does not affect its centromeric targeting, removal of the SANTA domain or the *C*-terminal region significantly impairs this localization (Fig. 2, Supplementary Fig. S6). This indicates that centromeric targeting of βKNL2 primarily relies on the SANTA domain and *C*-terminal motifs, independent of the *N*-terminal region. Further specificity was explored using constructs with separate deletions of motifs I, II, and III. Results showed that motif-III (AA 229–249) in the *C*-terminus is essential for the correct localization of βKNL2, as its deletion causes the protein to mislocalize to the cytoplasm, similar to the complete deletion of the *C*-terminus. In contrast, deletions of motif-I and motif-II do not affect centromeric localization (Fig. 3, Supplementary Fig. S7). AlphaFold3 predicted structural changes in these motifs when βKNL2 forms a dimer or oligomer (Supplementary Fig. S16), suggesting these motifs contribute to structural organization and interactions.

Detailed analysis revealed that motif-III encompasses a SUMOylation site spanning residues 221–231, with lysine 229 representing the putative SUMO acceptor. This suggests that SUMOylation contributes to regulating βKNL2 localization or interaction dynamics. *In vitro* SUMOylation assays confirmed that βKNL2 can be modified by SUMO1 at this site. Complete deletion of motif-III (AA229-249) caused loss of nucleoplasmic localization and increased cytoplasmic accumulation, while centromeric localization was retained, closely resembling the effect of *C*-terminal deletion. However, deleting the highly conserved region of motif-III (AA 237–249) while retaining the SUMOylation region led to partial restoration of nucleoplasm localization. These results indicate that the integrity of the entire motif-III is important for proper nuclear and centromeric localization, and that the SUMOylation site alone is not sufficient to fully restore targeting.

SUMOylation is known to regulate protein-protein interactions, subcellular localization, and stability, and is broadly recognized as essential for kinetochore assembly and centromere maintenance [41, 42, 48, 49]. Deletion of motif-III, which harbors the SUMO-acceptor site, mislocalizes βKNL2, indicating a SUMO-dependent step in nuclear targeting or retention. How SUMOylation promotes βKNL2 localization remains to be elucidated.

In human cells, depletion of the SUMO protease SENP6 leads to hyper-SUMOylation and mislocalization of core kinetochore proteins such as CENP-C, CENP-I, and M18BP1/KNL2 [50–52]. While the mechanistic outcome in plants may differ, these observations highlight that altered SUMOylation status can disrupt kinetochore integrity. SUMOylated kinetochore proteins are known to interact with deSUMOylases such as SENP6 (Ulp2 in yeast) and the segregase p97/Cdc48, which together safeguard centromere identity by regulating CENP-A deposition and kinetochore integrity. Given these parallels, we propose that SUMOylation at motif-III could be one of multiple regulatory inputs stabilizing βKNL2 at the nucleus and centromeres. Definitive testing of this model will require *in vivo* SUMOylation site mutagenesis and functional assays in plants.

Our study identified specific regions essential for the centromeric targeting of βKNL2, prompting further investigation into the mechanisms of this targeting process and the role of βKNL2 in kinetochore assembly. French and Straight [24] showed that human M18BP1/KNL2 localizes to centromeres during metaphase by binding to the CENPC-motif containing CENP-C protein using a conserved SANTA domain. Notably, in plants, both αKNL2 and CENP-C contain the CENPC-motif. In reproductive tissues like pollen and developing seeds, but not in embryos, centromeric localization of βKNL2 relies on αKNL2, indicating a tissue-specific recruitment mechanism (Supplementary Fig. S11).

In humans, M18BP1/KNL2 primarily forms homodimers through interaction with the Mis18α&β 4:2 hexamer, mediated by the *N*-terminal region [18, 44, 53]. However, recent evidence shows that artificial dimerization of M18BP1 can bypass Mis18α/β, suggesting that M18BP1 can also dimerize independently [47]. In plants, no Mis18α or Mis18β have been identified, but instead, two KNL2 variants are present. Thus, different compositions of KNL2 complexes can be expected in plants.

Our structural predictions and experimental evidence show that βKNL2 associates with αKNL2 and is capable of homomeric interactions. AlphaFold models suggest that βKNL2 dimerization is mediated through its pre-SANTA and SANTA domains, resembling the dimer interface seen in *Xenopus* and human KNL2 [18, 24, 47]. These predictions are further supported by BiFC and co-IP assays, which demonstrate an association between βKNL2 and αKNL2 in planta. Further insight into the interaction interface was obtained using Y2H analysis, where truncated constructs revealed that the SANTA-containing regions are sufficient to mediate interaction, whereas full-length proteins did not interact. Specifically, interaction between αKNL2(1–118) and βKNL2(50–154), but not with the extended βKNL2(50–218), indicates that the interface is confined to the SANTA-containing region and depends on its accessibility. These findings suggest that the SANTA domain represents a primary interaction interface, which may be partially masked in full-length proteins, likely due to intramolecular folding or domain packing that limits exposure of the interaction surface. In addition, the absence of plant-specific cofactors or post-translational modifications in the yeast system may further restrict the formation of this interaction.

Since deletion of the proline-rich *N*-terminus did not affect the centromeric localization of βKNL2, it is conceivable that it might enhance oligomerization and complex stability. The *N*-terminal prolines are predicted to be phosphorylated, indicating possible regulatory control through post-translational modifications. In vertebrates, CDK1 regulates M18BP1/KNL2 centromere localization through dimerization and oligomerization [18, 24]. Interestingly, we identified a conserved region just before the SANTA (TPV/IK), recognized as a CDK motif in the *N*-terminus (Supplemental File.3). Furthermore, AlphaFold3 prediction that βKNL2 and αKNL2 bind to the nucleosome also shows that the SANTA domains and pre-SANTA conserved AA (S_14_F_15_Q_16_ in αKNL2 & L_95_F_96_S_97_ in βKNL2) form an intermonomeric β-sheet. All these findings underscore the role of the βKNL2 SANTA domain in centromere targeting and dimerization with αKNL2.

**Figure 5.**
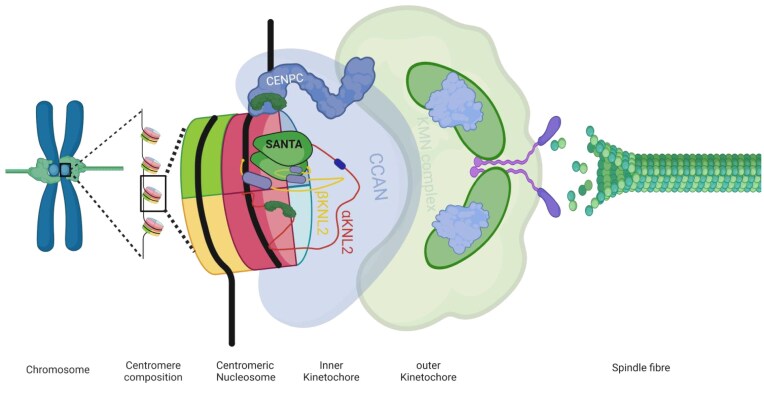
Graphical model of βKNL2 dimerization supporting centromere assembly and kinetochore platform. This graphical representation illustrates how βKNL2 dimerizes to facilitate the assembly and function of the centromere and kinetochore structures. βKNL2 forms dimers with αKNL2 and interacts with the centromeric nucleosome. These interactions are mediated through the SANTA domain and conserved motifs in the *C*-terminus, which are essential for stabilizing the kinetochore platform and promoting efficient centromere assembly. Created with BioRender.com

The inner kinetochore consists of structural and DNA-binding proteins that typically bind to centromeres [4, 54]. αKNL2 can bind centromeric DNA *in vitro* and *in vivo* [23], and its centromeric localization requires the presence of DNA-binding regions in addition to the CENPC-k motif [43]. Here we show that βKNL2 can bind to centromeric DNA independent of αKNL2, supporting βKNL2’s involvement in inner kinetochore dynamics and chromatin interactions. In different organisms, M18BP1/KNL2 and CENP-C play common roles in different cell cycle stages, such as CENP-A loading and kinetochore assembly. The CENP-C cupin domain, a feature absent in plants, oligomerizes for kinetochore assembly [55]. Highlighting these unique features of plants with two KNL2s and their ability to dimerize raises questions about their adaptation to a different kinetochore assembly platform. We propose that βKNL2, together with αKNL2 and/or CENP-C, facilitates or supports the kinetochore assembly platform (Fig. 5). Evidence such as the consistent presence of βKNL2 at centromeres throughout the cell cycle, reduced or abolished CENP-A/CENH3 loading onto centromeres when βKNL2 expression is disrupted, and its interaction with αKNL2,always exposing the negative pocket of βKNL2 and the site we identified for SUMOylation (except when predicted with SUMO, which binds there) all support its role in the kinetochore assembly platform [26].

## Supplementary Material

gkag605_Supplemental_Files

## Data Availability

Mass spectrometry proteomics data have been deposited in PRIDE (https://www.ebi.ac.uk/pride/archive) under accession number PXD067361.

## Notes

Present address: Centre of Plant Structural and Functional Genomics, Institute of Experimental Botany, Czech Academy of Sciences, Šlechtitelů 31, Olomouc 77900, Czech Republic
