# Supplementary material for: Structural basis of βKNL2 centromeric targeting mechanism and its role in plant-specific kinetochore assembly
Source: Nucleic Acids Res. 2026 Jun 25;54(12):gkag605. doi: 10.1093/nar/gkag605 (PMC13294675; doi:10.1093/nar/gkag605)
Supplement: gkag605_Supplemental_Files [file gkag605_supplemental_files.zip › supp figs.pdf]

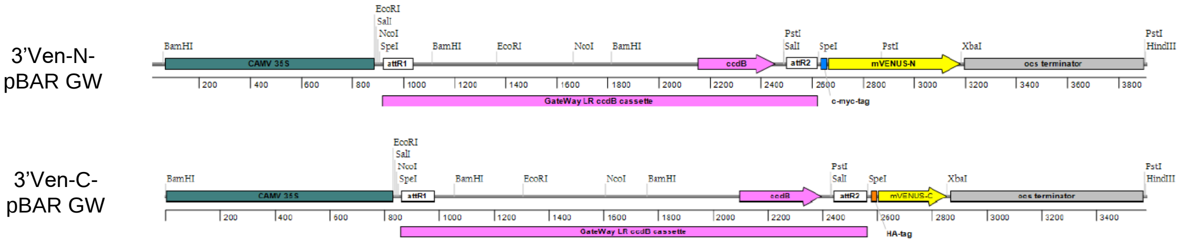

Supplementary Figure S1. Schematic diagram of BiFC expression vector construction. The construction of BiFC expression vectors using the pPZP200BAR binary vector. The CAMV35S promoter and OCS terminator were integrated via overlap extension PCR. The GateWay LR ccdB cassette along with mVENUS-N or mVENUS-C, each tagged with cMYC or HA respectively, were amplified by PCR and subsequently cloned into the pPZP200BAR vector between the CAMV35S promoter and OCS terminator.

βKNL2
MTTTRAKSKFQSLSACRFTPLPEPNTSPSTYSKTL
PKPNSSPGTDGTFPTPFPLAVITPIKTLKSVTLSD
WWLTKKGKDLCIKGFESNGASGVRLFSSGTISKRH
ESTTLEAIDGITISINGFINRSRCLENGISIEVCN
RFRLGFPYDWEDYNEEEEEKKKKNVDISFDDIPVN
RYQDLYSLEGCLKDKILDDVVGSLRDLVCQKSDKA
CEKSRVGDVDDDDDDDDDKSLVSRVVGVKTRGMLR
RREEYEASIGKRVATMSGKRVVTVSKKKNRRRSFG
W

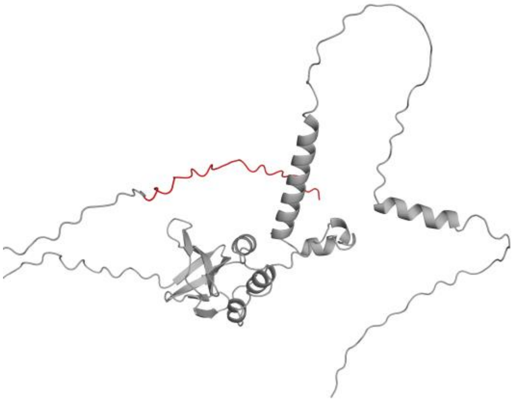

αKNL2
MTEPNLDEDGSKSSFQKTVVLRDWWLIKCPKEFEG
KQFGVAGFEESVETRAMRVFTSSPITKALDVFTLL
ASDGIYITLRGFLNKERVLKNGFNPEISREFIFGF
PPCWERVCNSCFEGDSFGTDVNTVPSTIEKACPPI
LSPCKYSNRNLKDNPAESREKSNVTETDIAEINDK
GGSGARDIKTARRRSLHLQIKRILESSKVRKTAND
GDHGSEFLNTAKRGDVERDGCEVINNEDSEWKLDE
SEVQNLCNDGDNGSEGFIKAKSSDVEKDKSEAIDN
DVISPAVGSGIKHTGADNVDKVTSASATGESLTSE
QQNGLLVTTASPHSLLKDLAKSSKPEKKGISKKSG
KILRSDDNVVDPMNYSGTKVKSAENKRKIDASKLQ
SPTSNVAEHSKEGLNNAKSNDVEKDVCVAINNEVI
SPVKGFGKRLSGTDVERLTSKNATKESLTSVQRKG
RVKVSKAFQDPLSKGKSKKSEKTLQSNSNVVEPMN
HFRSEAEEAEENLSWEKIKRKIDFDVEVTPEKKVK
QQKTNAASTDSLGQKRSRSGRVLVSSLEFWRNQIP
VYDMDRNLIQVKDGSETNSAPSKGKGSDSRKRRNL
KIK

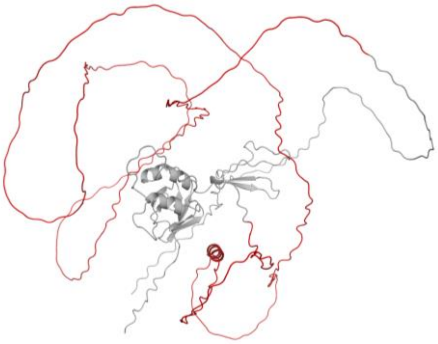

CenH3
MARTKHRVTRSQPRNQTDAAGASSSQAAGPTTTPT
RRGGEGGDNTQQTNPTTSPATGTRRGAKRSRQAMP
RGSQKKSYRYRPGTVALKEIRHFQKQTNLLIPAAS
FIREVRSITHMLAPPQINRWTAEALVALQEAAEDY
LVGLFSDSMLCAIHARRVTLMRKDFELARRLGGKG
RPW

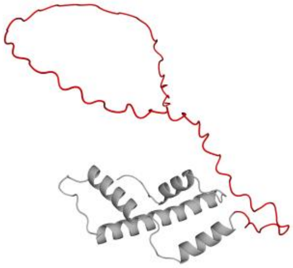

H2AW6
MESTGKVKKAFGGRKPPGAPKTKSVSKSMKAGLQF
PVGRITRFLKKGRYAQRLGGGAPVYMAAVLEYLAA
EVLELAGNAARDNKKSRIIPRHLLLAIRNDEELGK
LLSGVTIAHGGVLPNINSVLLPKKSATKPAEEKAT
KSPVKSPKKA

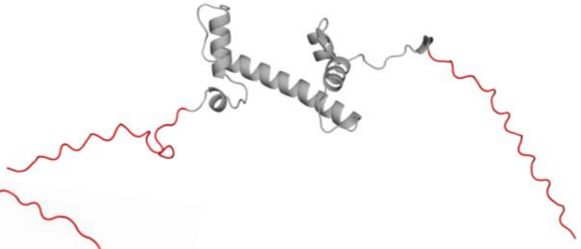

H2BHTB9_A
MAPRAEKKPAEKKPAAEKPVEEKSKAEKAPAEKKP
KAGKKLPKEAGAGGDKKKKMKKKSVETYKIYIFKV
LKQVHPDIGISSKAMGIMNSFINDIFEKLASESSK
LARYNKKPTITSREIQTAVRLVLPGELAKHAVSEG
TKAVTKFTSS

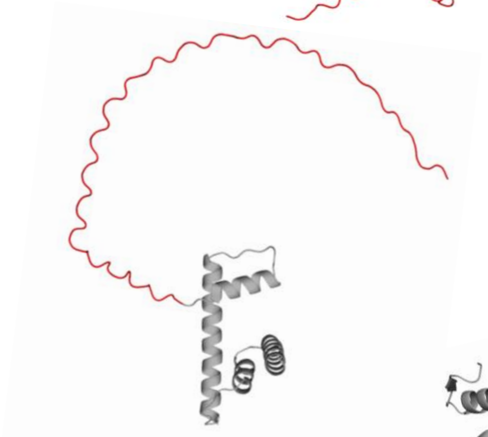

H4_A
MSGRGKGGKGLGKGGAKRHRKVLRDNIQGITKPAI
RRLARRGGVKRISGLIYEETRGVLKIFLENVIRDA
VTYTEHARRKTVTAMDVVYALKRQGRTLYGFGG

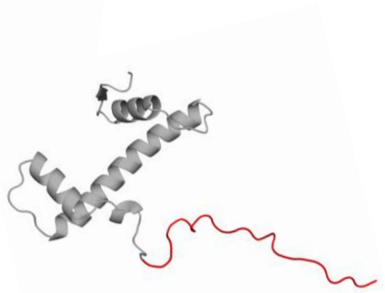

Supplementary Figure S2. Neglected regions of the monomers in the prediction and modelling of βKNL2 and αKNL2 with the octameric histones. The sequences for each monomer are in the left column, with neglected regions in red, and the correspondent predicted structures are in the right column

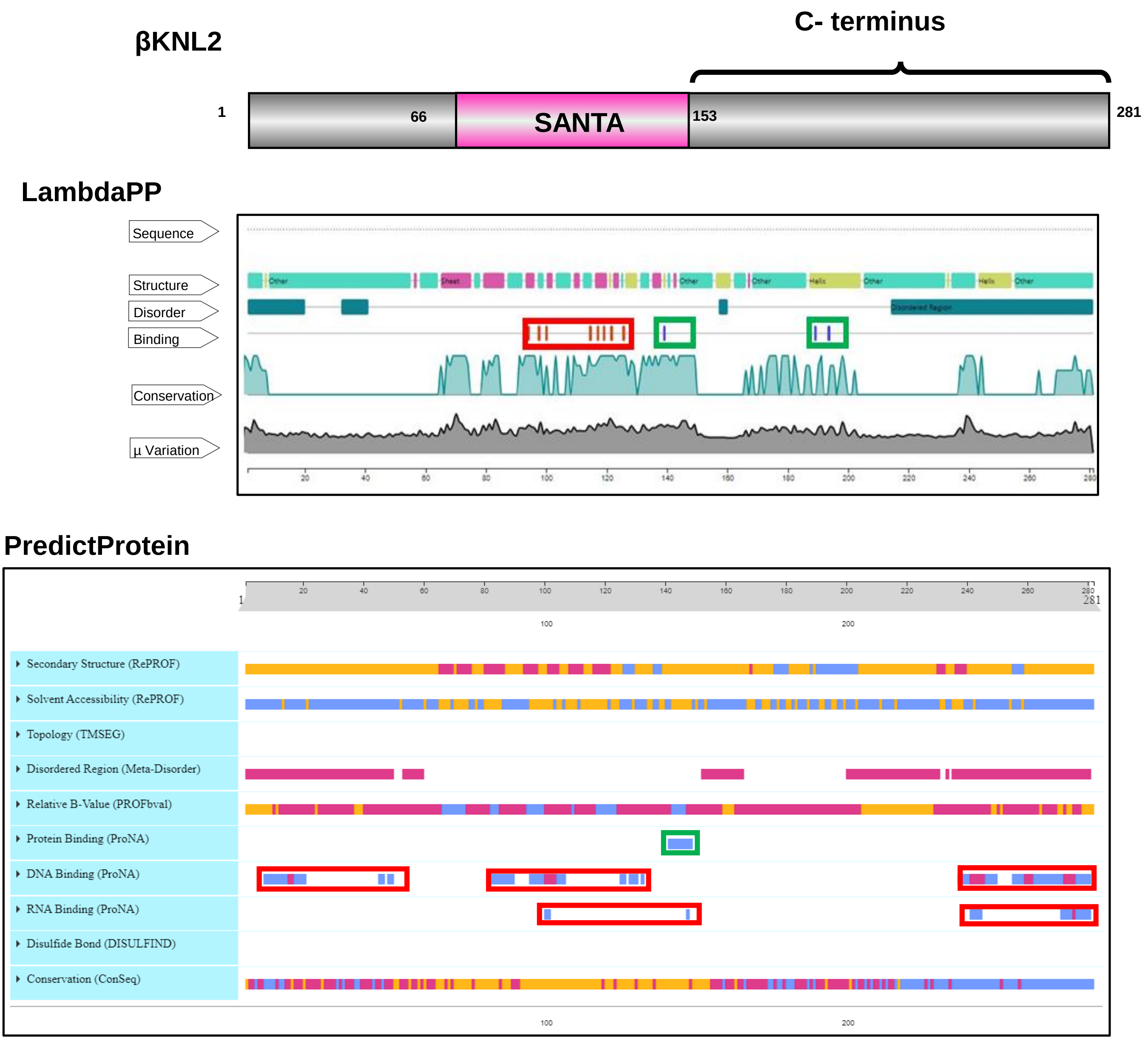

Supplementary Figure S3. *In silico* prediction of protein-DNA and protein-protein interaction sites of βKNL2. *In silico* analysis of the βKNL2 protein, highlighting potential interaction sites for proteins (indicated in green) and nucleic acids (indicated in red). Computational predictions using LambdaPP and PredictProtein tools suggest that the SANTA domain and C-terminus of βKNL2 have the potential to interact with other proteins and nucleic acids, respectively. Each color-coded box corresponds to regions within the βKNL2 structure predicted to facilitate these molecular interactions.

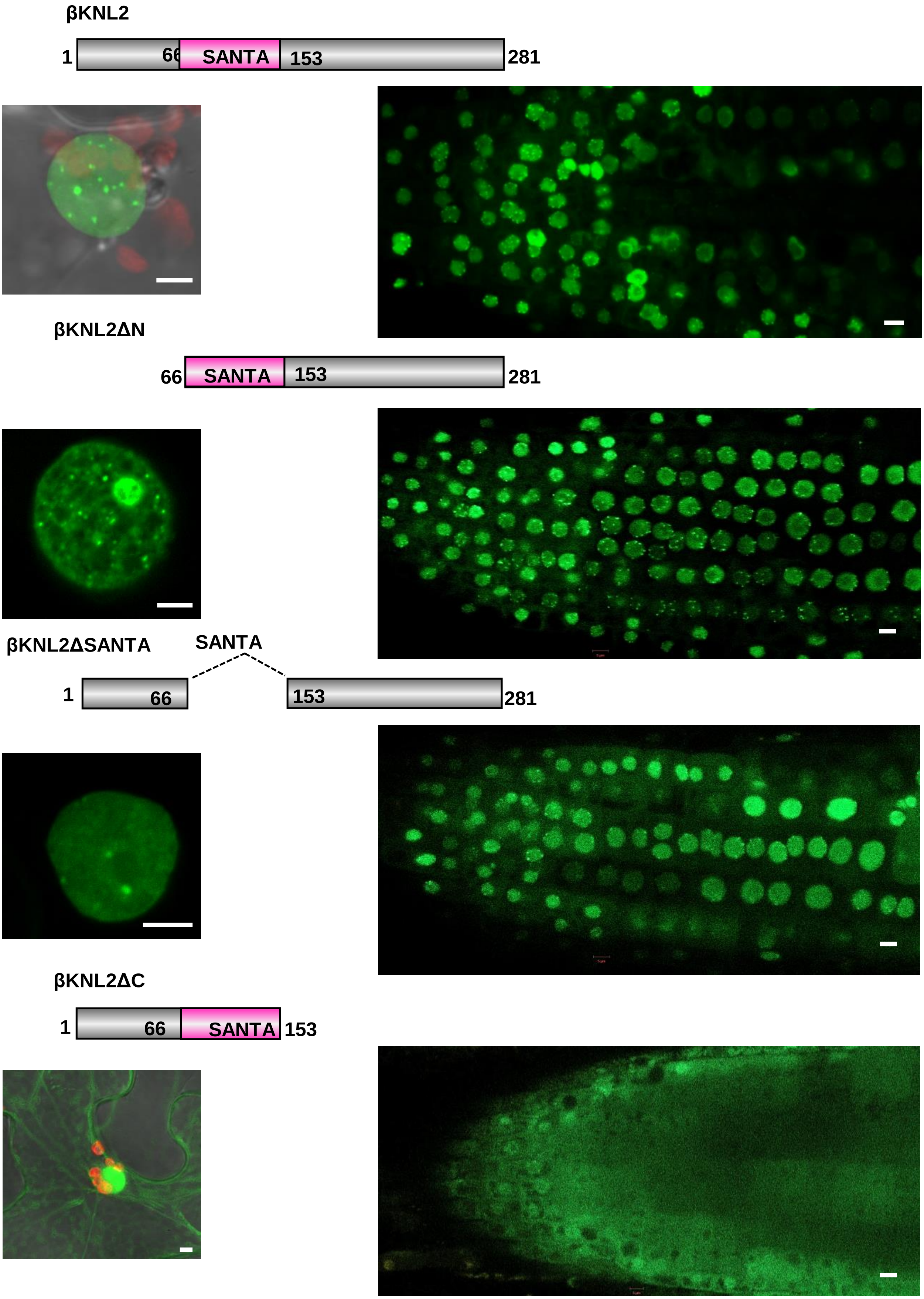

Supplementary Figure S4: Localization patterns of EYFP-tagged βKNL2 truncated variants in *N. benthamiana* and *A. thaliana.* Left panels showing localization patterns of truncated βKNL2 variants when transiently expressed in *N. benthamiana* leaves. The right panels display a representative root tip from *Arabidopsis*, providing a stable expression context from which main Figure 1C was derived. The full length βKNL2 and βKNL2ΔN exhibit centromere-like localization patterns marked by distinct nuclear dots. Whereas βKNL2ΔSANTA showed reduced centromeric targeting compared to control. In contrast, the βKNL2ΔC variant shows cytoplasmic and nucleoplasmic localization, indicating a mis-localization pattern compared to the full-length and other truncated variants. Scale bars represent 5μm.

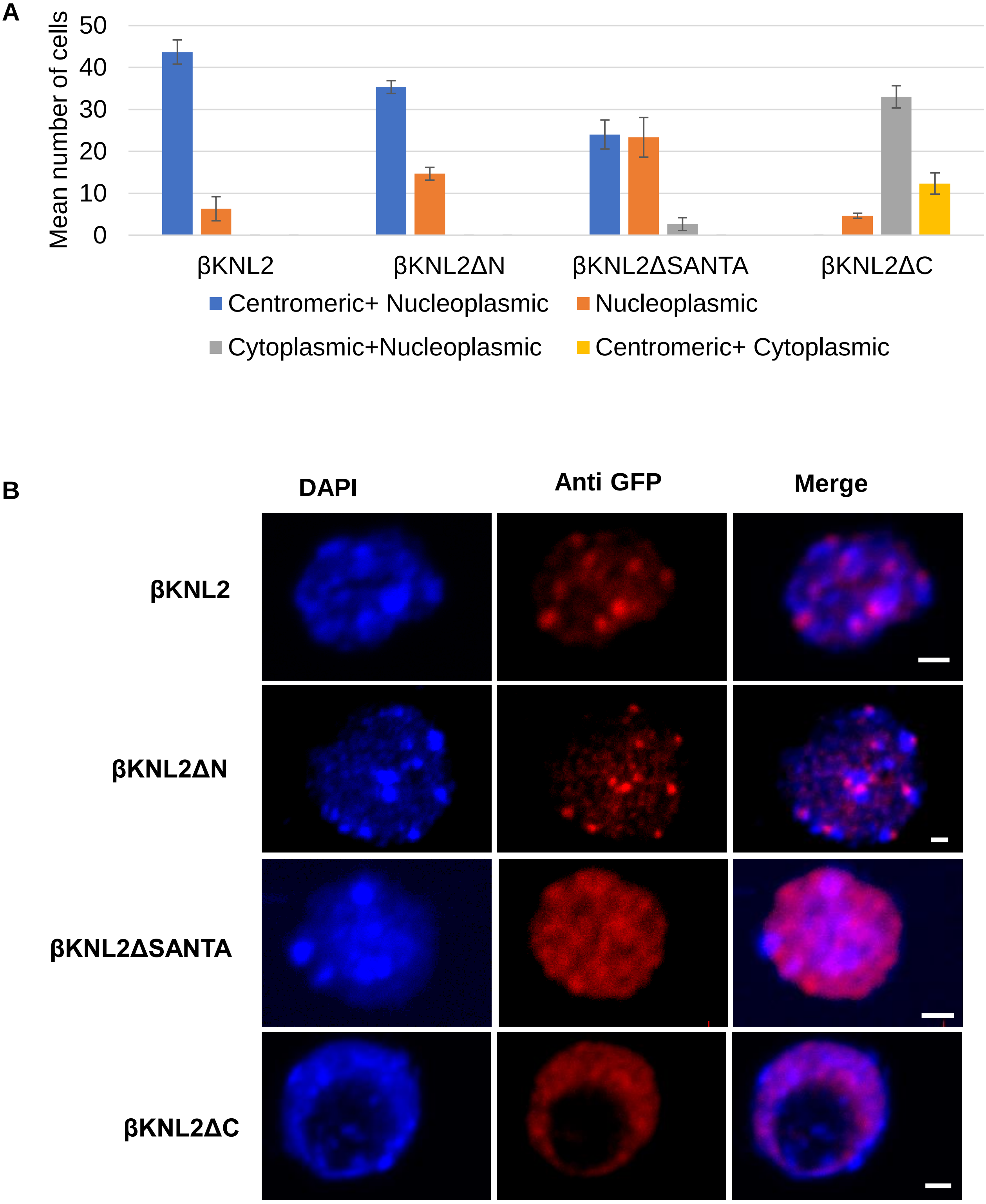

Supplementary Figure S5. Quantitative and immunolocalization analysis of βKNL2 and its truncation variants. (A) Fluorescence patterns of transiently expressed βKNL2 truncation constructs fused to EYFP in *Nicotiana benthamiana*. Four distinct fluorescence localization patterns were quantified: nucleoplasmic + centromeric, nucleoplasmic, cytoplasmic + nucleoplasmic, and cytoplasmic + centromeric. The frequency of nuclei exhibiting each pattern was determined for each construct based on three independent infiltrations. 50 nuclei were analyzed from *N. benthamiana* leaf of each infitration. (B) Immunostaining was performed using an anti-GFP antibody (red) to detect βKNL2-EYFP fusion proteins in stably transformed *Arabidopsis* lines. Nuclei were counterstained with DAPI (blue) to visualize chromocenters, which correspond to centromeric heterochromatin. Merged images (right column) show co-localization of full-length βKNL2-EYFP and βKNL2ΔN-EYFP with DAPI-dense chromocenters, indicating centromeric localization. In contrast, βKNL2ΔSANTA and βKNL2ΔC variants exhibit diffuse nucleoplasmic and non-specific signals, lacking clear centromeric enrichment. These results suggest that both the SANTA domain and the C-terminal region contribute to proper centromere targeting. Scale bar: 1 µm

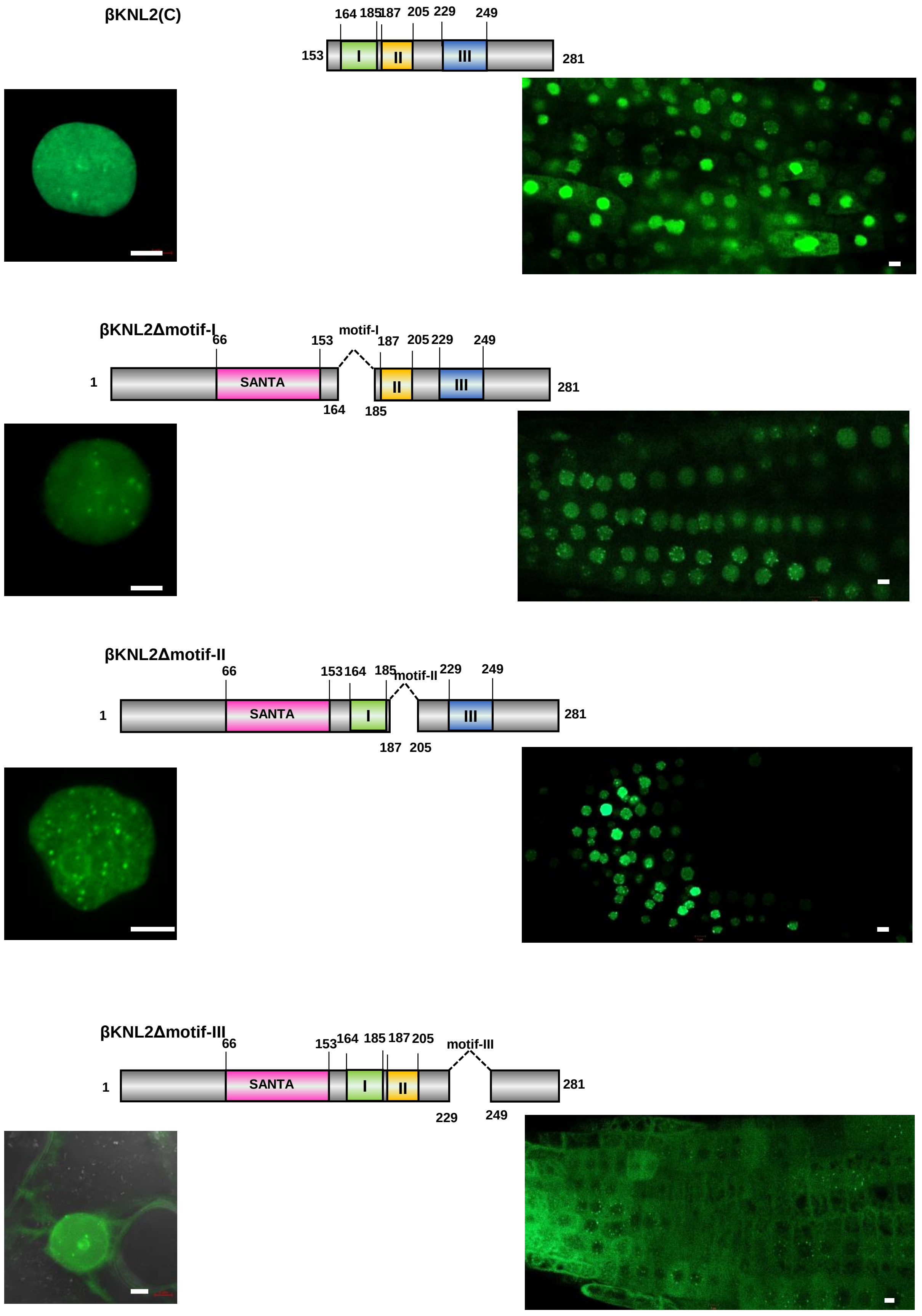
βKNL2(C)
164 185 187 205 229 249
153 I II III 281
βKNL2Δmotif-I
66 153 motif-I 187 205 229 249
1 SANTA II III 281
164 185
βKNL2Δmotif-II
66 153 164 185 motif-II 229 249
1 SANTA I III 281
187 205
βKNL2Δmotif-III
66 153 164 185 187 205 motif-III
1 SANTA I II 281
229 249

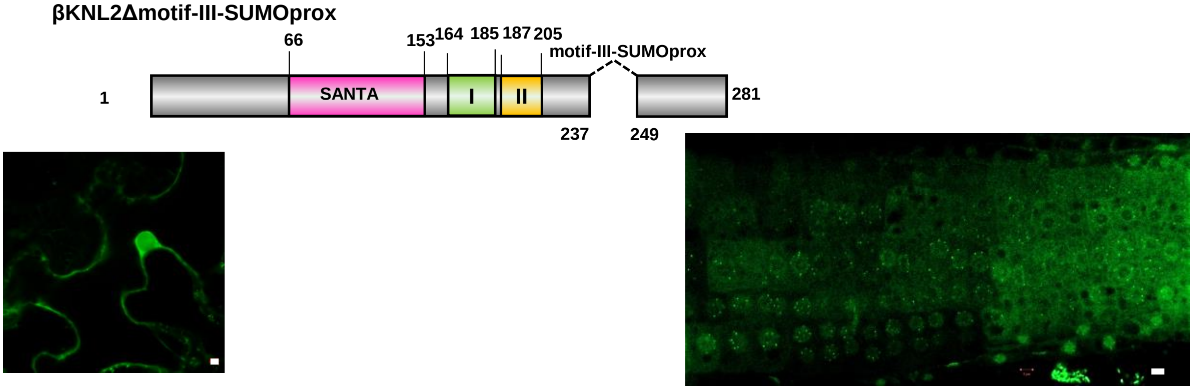

Supplementary Figure S6: Localization patterns of EYFP-tagged βKNL2 C-terminal truncated variants in *N. benthamiana* and *A. thaliana.* Left panels showing transient expression patterns of truncated βKNL2 variants βKNL2(C), βKNL2Δmotif-I, βKNL2Δmotif-II, and βKNL2Δmotif-III in tobacco leaves. The right panel of the figure shows a representative root tip of *Arabidopsis* displaying stable expression of these constructs, from which main Figures 2C were extracted. The deletion of motifs I and II retains the localization pattern typical to the full-length βKNL2. In contrast, deletion of motif-III leads to mis-localization of βKNL2Δmotif-III to the cytoplasm. Scale bar: 5 µm.

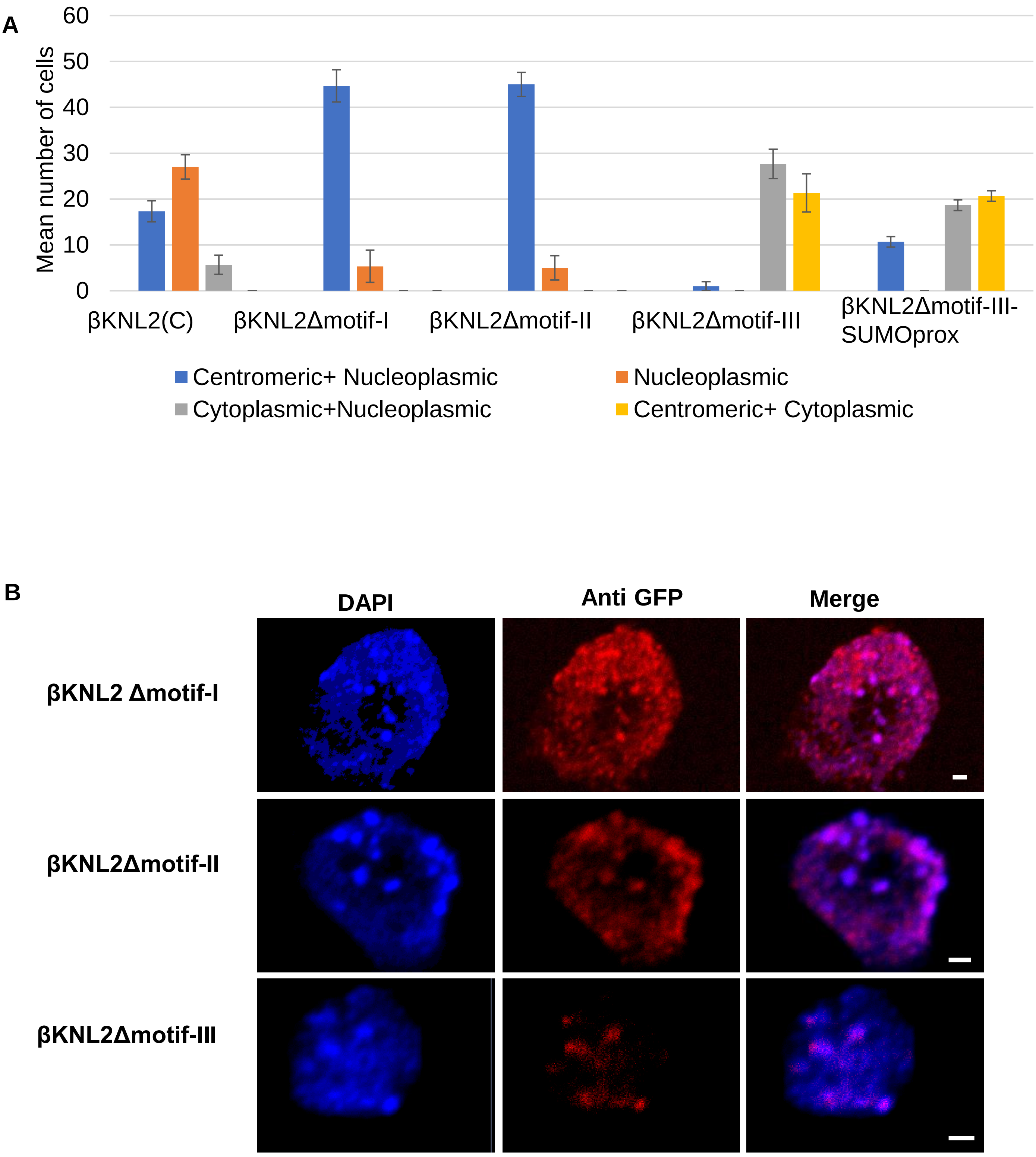

Supplementary Figure S7. Quantitative and immunolocalization analysis of βKNL2 C-terminal truncation variants. (A) Fluorescence patterns of transiently expressed βKNL2 C-terminal truncation constructs fused to EYFP in *Nicotiana benthamiana*. Four distinct fluorescence localization patterns were quantified: nucleoplasmic + centromeric, nucleoplasmic, cytoplasmic + nucleoplasmic, and cytoplasmic + centromeric. The frequency of nuclei exhibiting each pattern was determined for each construct based on three independent infiltrations. 50 nuclei were analyzed from *N. benthamiana* leaf of each infitration. (B) Immunostaining was performed using an anti-GFP antibody (red) to detect βKNL2Δmotif-I, βKNL2Δmotif-II, and βKNL2Δmotif-III EYFP-tagged fusion proteins in stably transformed *Arabidopsis* lines. Nuclei were counterstained with DAPI (blue) to mark chromocenters corresponding to centromeric heterochromatin. Merged images (right column) illustrate co-localization of βKNL2Δmotif-I-EYFP and βKNL2Δmotif-II-EYFP localize to both the nucleoplasm and DAPI-dense chromocenters, indicating retained centromeric localization. In contrast, βKNL2Δmotif-III-EYFP signals are confined to chromocenters and reduced in the nucleoplasm, suggesting impaired nuclear distribution. Scale bar: 1 µm

ELM prediction of SUMO sites

FPLAVIT<br>RVVTVS	52-58 [A]<br>265-270 [A]	-<br>-	Motif for the parallel beta augmentation mode of non-covalent binding to SUMO protein.
DDDDDDDDKSL<br>DDDDDDDKSL<br>DDDDDDKSL<br>DDDDDKSL<br>DDDDKSL<br>DDDKSL	221-231 [A]<br>222-231 [A]<br>223-231 [A]<br>224-231 [A]<br>225-231 [A]<br>226-231 [A]	-<br>-<br>-<br>-<br>-<br>-	Inverted version of SUMOylation motif recognized for modification by SUMO-1

GPS-SUMO 2.0 prediction of SUMO sites

266-270	ATMSGKRVVTVSKKKNRRR	0.8959	0.5	SUMO interaction	Pred.

Supplementary Figure S8: Predicted SUMOylation sites overlap with motif-III of βKNL2. ELM and GPS-SUMO 2.0 bioinformatics tools predict putative SUMOylation sites in a region of βKNL2 spanning AA 221-231. This region overlaps with motif-III, which extends from AA 229 to 249. In addition, both ELM and GPS-SUMO 2.0 identify a SUMO interaction motif spanning residues 266 to 270.

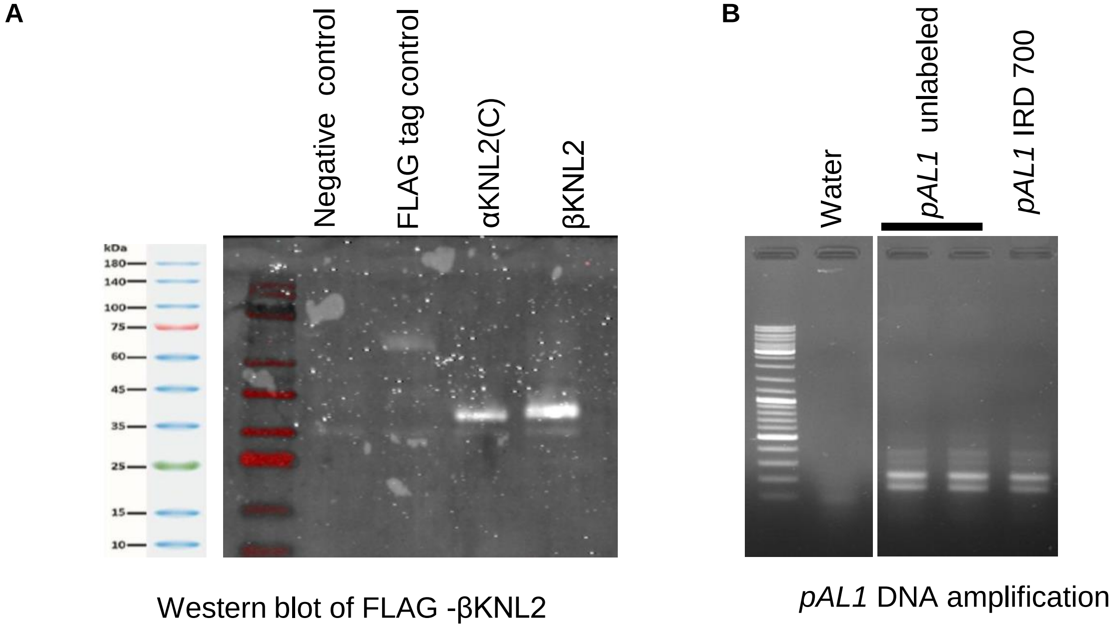

Supplementary Figure S9: Expression and verification of FLAG-βKNL2 and FLAG-αKNL2(C), and PCR amplification of the *pAL1* DNA. (A) Western blot analysis confirming the expression of FLAG-tagged αKNL2(C) and βKNL2 proteins in TNT SP6 High Yield Wheat Germ system (Promega) using anti-FLAG antibodies. Despite their different expected sizes, αKNL2(C) (26.36 kDa) and βKNL2 (31.56 kDa) proteins exhibit similar migration patterns, which may be attributed to post-translational modifications. (B) The PCR amplification of the *pAL1* centromeric repeat from *Arabidopsis* genomic DNA.

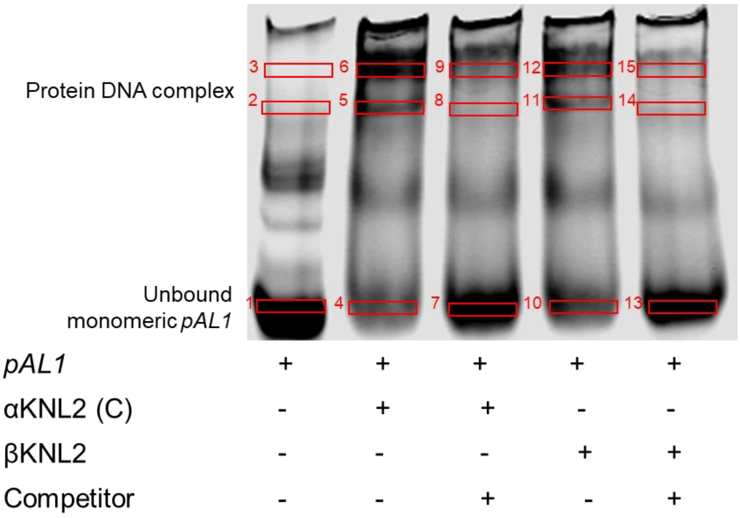

Region	Area	Mean intensity	SD	Max	Min
1	700	238	0	238	238
2	700	44.414	14.619	91	23
3	700	15.909	6.663	40	6
4	700	157.496	12.512	187	119
5	700	178.08	27.398	238	136
6	700	229.044	13.174	238	187
7	700	238	0	238	238
8	700	81.893	11.726	119	57
9	700	134.099	17.897	204	102
10	700	180.227	22.457	238	119
11	700	155.55	38.813	238	108
12	700	186.123	31.612	238	136
13	700	238	0	238	238
14	700	63.003	10.367	85	40
15	700	78.736	15.102	136	51

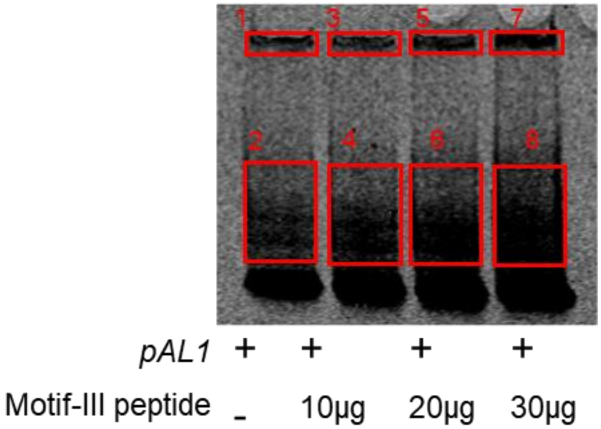

Region	Area	Mean intensity	SD	Max	Min
1	650	84.531	30.555	132	8
2	650	84.295	27.414	132	7
3	650	93.812	31.425	132	9
4	650	108.172	28.812	132	12
5	3550	90.986	23.600	132	6
6	3550	98.911	27.595	132	8
7	3550	106.473	25.517	132	9
8	3550	117.577	18.989	132	15

Supplementary Figure S10. Quantitative analysis of DNA–protein interaction using ImageJ. Top panel: EMSA gel showing DNA-protein complex shifts in the presence and absence of competitor DNA. Red boxes indicate the defined regions used for quantification. Corresponding table shows the mean intensity, standard deviation (SD), and pixel range (Max, Min) for each selected band region. A decrease in band shift intensity was observed with addition of competitor DNA. **Bottom panel:** EMSA analysis showing increasing concentrations of the βKNL2 motif-III peptide with constant DNA levels. Quantification of the shifted bands reveals a concentration-dependent increase in signal intensity.

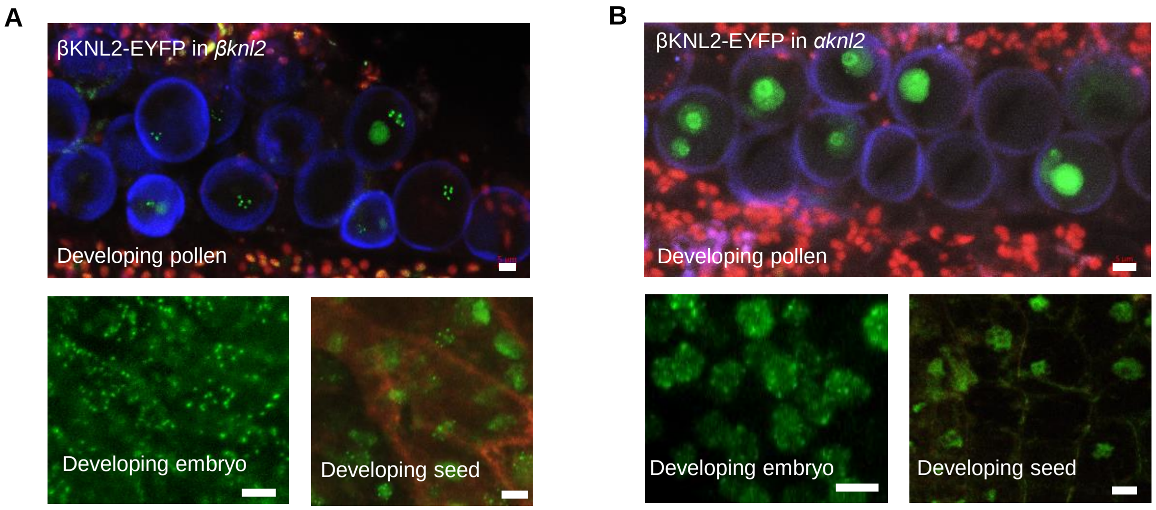

Supplementary Figure S11: Disrupted centromeric localization of βKNL2-EYFP in *αknl2* mutants. (A) *Arabidopsis βknl2* transformants expressing βKNL2::βKNL2-EYFP serving as controls showed βKNL2-EYFP signals localized in both the centromeres and nucleoplasm within the nuclei of developing pollen, embryos, and seeds. (B) In *αknl2* mutant transformants, βKNL2-EYFP was primarily observed in the nucleoplasm of developing pollen and seeds. Notably, in developing embryos, βKNL2-EYFP targeted to the centromeres, similar to *βknl2* mutant transformants. Scale bar: 5µm.

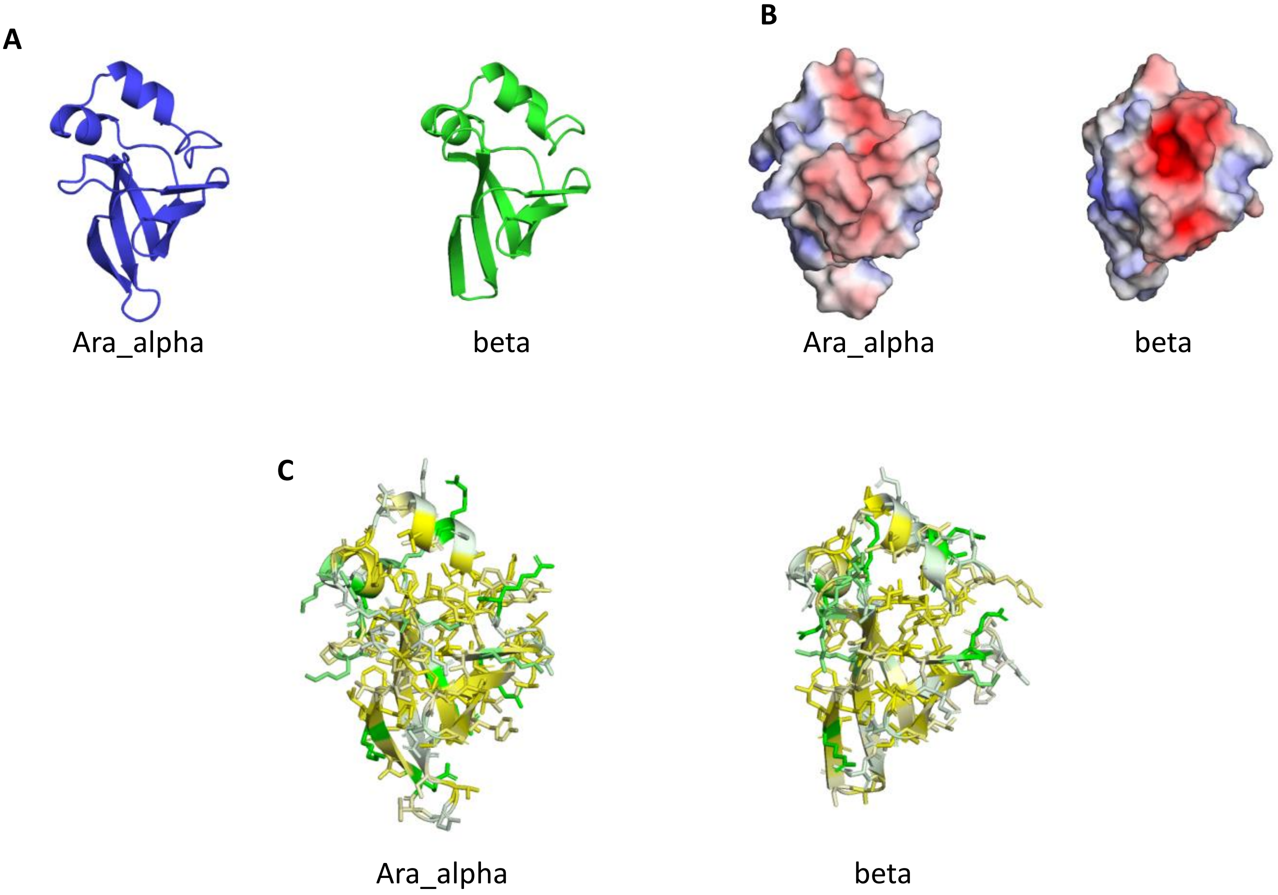

Supplementary Figure S12: Analysis of *Arabidopsis* SANTA domains. In each panel, we show SANTA domains of αKNL2 and βKNL2. After superposing SANTA domains, we oriented them to show the negatively charged pocket from βKNL2's SANTA domain. Three panels show different properties: (A) cartoon mode shows tertiary structure. αKNL2's and βKNL2's SANTA domain are colored in blue and green, respectively. (B) Surface mode with electrostatic charge surface distribution colored from most negatively charge (red) to most positively charged (blue). (C) Cartoon and sticks mode showing each residue colored by hydrophobicity values taken from Eisenberg scale (ref), colored from most hydrophilic (green) to most hydrophobic (yellow).

Contacts between bKNL2 and aKNL2

#	Model (chains of bKNL2 and aKNL2)	bKNL2	aKNL2	Sumo1	nucleosome
A	fold_abknl2_model_0.cif (A, B)	1	1	0	0
B	fold_two_bknl2_and_two_sumo1_two_aknl2_nucleosome_model_0.cif (A, E)	2	2	2	1
C	fold_two_bknl2_and_two_sumo1_two_aknl2_nucleosome_model_0.cif (B, F)	2	2	2	1
D	fold_two_bknl2_one_sumo1_and_aknl2_model_0.cif (A, D)	2	1	1	0
E	fold_two_a_bknl2_nucleosome_with_pal1_model_0.cif (A, E)	2	2	0	1
F	fold_two_a_bknl2_nucleosome_with_pal1_model_0.cif (B, F)	2	2	0	1
G	fold_two_aknl2_one_bknl2_model_0.cif (A, B)	1	2	0	0
H	fold_two_bknl2_two_sumo1_and_aknl2_model_0.cif (A, E)	2	2	2	0
I	fold_two_bknl2_oneaknl2_model_0.cif (A, C)	2	1	0	0
J	fold_bknl2_aknl2n_model_0.cif (A, B)	1	1	0	0
K	fold_bknl2_aknl2n_aknl2c_model_0.cif (A, B)	1	1	0	0
L	fold_one_bknl2_one_sumo1_and_aknl2_model_0.cif (A, C)	1	1	1	0
M	fold_two_a_bknl2_nucleosome_with_dissordered_reagions_model_0.cif	2	2	0	1
N	fold_two_a_bknl2_nucleosome_with_dissordered_reagions_model_0.cif	2	2	0	1

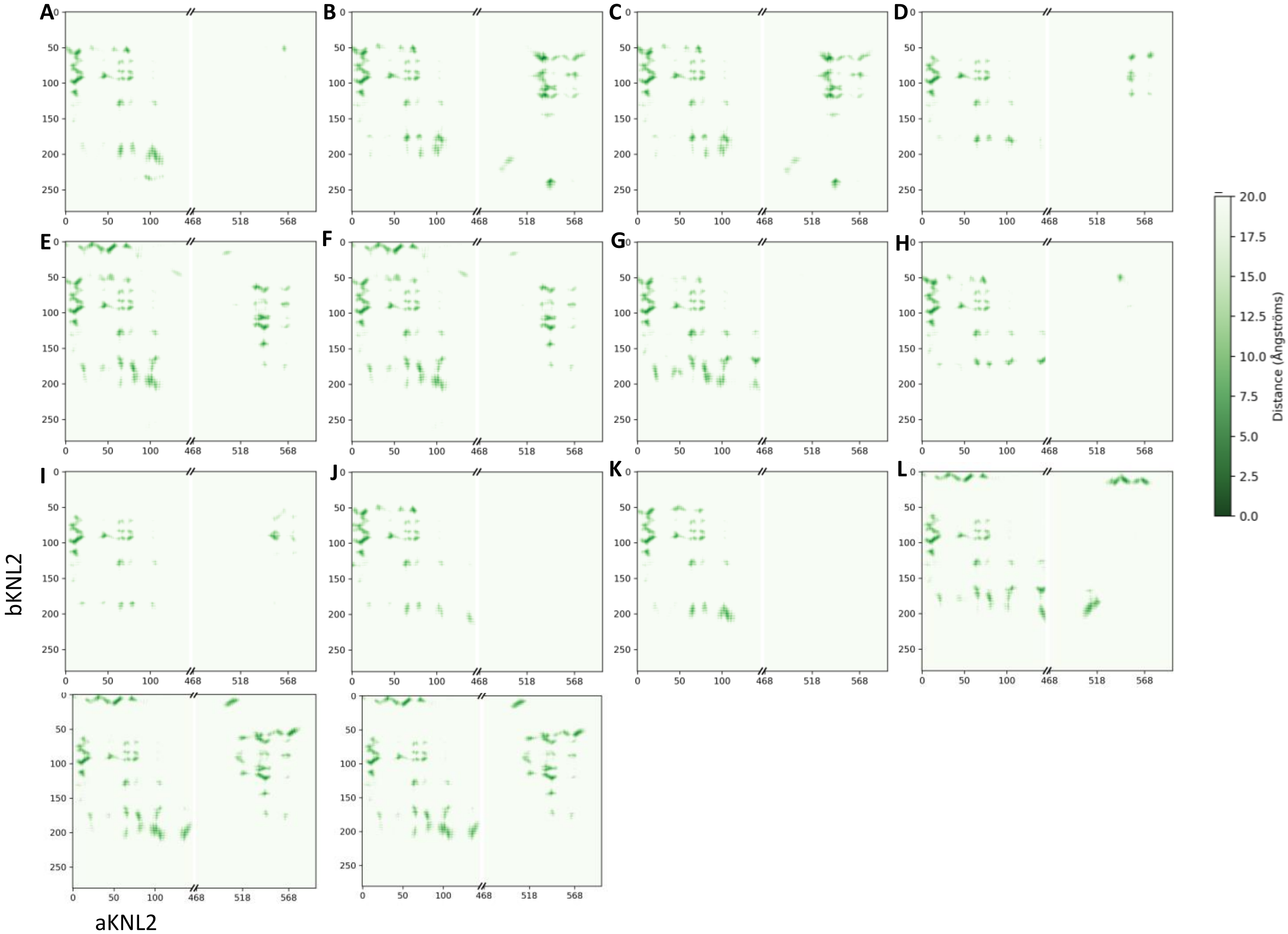

Supplementary Figure S13: Structural predictions with βKNL2 and αKNL2
The table lists all predictions where βKNL2 and αKNL2 are present, discriminating which and how many molecules. For each pair of βKNL2 and αKNL2 in the models there is one contact map below. βKNL2 residues are always in the y-axis and αKNL2 residues in the x-axis

Contacts between bKNL2 and bKNL2

#	Model (chains of bKNL2 and aKNL2)	bKNL2	aKNL2	Sumo1	nucleosome	Similar contact map group
A	fold_beta_dimer_model_0.cif (A, B)	2	0	0	0	1
B	fold_two_bknl2_oneaknl2_model_0.cif (A, B)	2	1	0	0	2
C	fold_two_beta_one_cenh3_model_0.cif (A, B)	2	0	0	(1 CenH3)	3
D	fold_two_bknl2_one_sumo1_and_aknl2_model_0.cif (A, B)	2	1	1	0	4
E	fold_two_bknl2_two_sumo1_and_aknl2_model_0.cif (A, B)	2	2	2	0	4

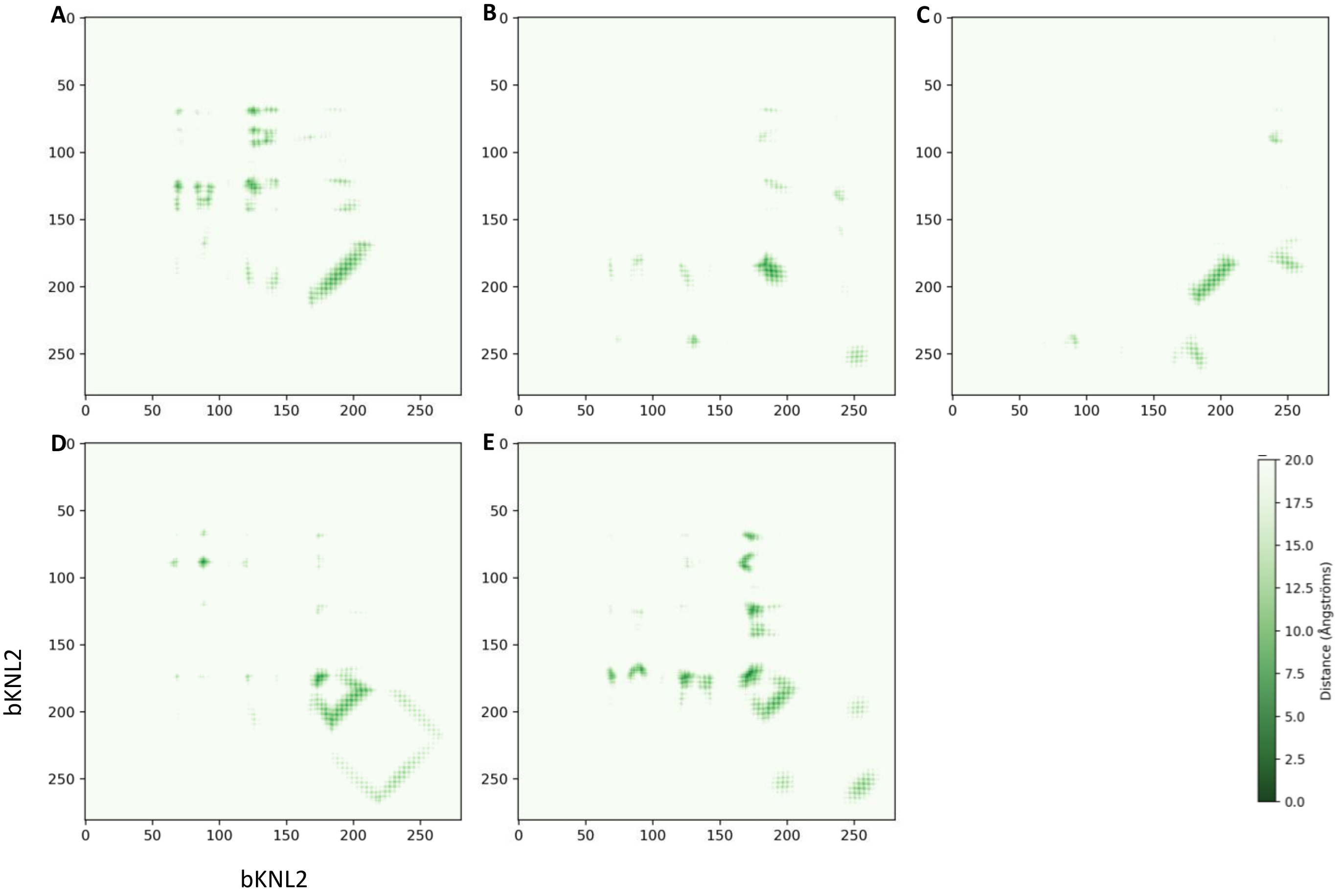

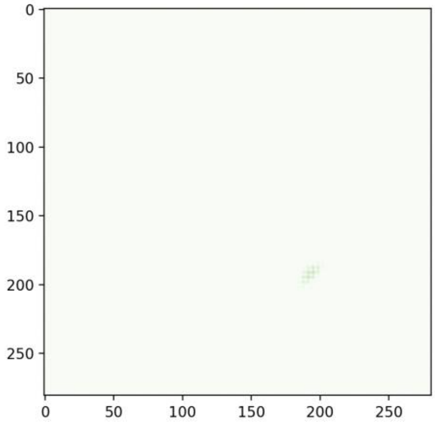

Supplementary Figure S14: Structural predictions with two molecules of βKNL2

The table lists all predictions where two molecules of βKNL2 are present, discriminating which and how many molecules. For each pair of βKNL2 molecules in the models there is one contact map below. The average of all contact maps is showed in the bottom.

Contacts between bKNL2 and SUMO1

#	Model (chains of bKNL2 and SUMO1)	bKNL2	aKNL2	Sumo1	nucleosome	Similar contact map group
A	fold_two_bknl2_and_two_sumo1_two_aknl2_nucleosome_model_0 (A, M)	2	2	2	1	1
B	fold_two_bknl2_and_two_sumo1_two_aknl2_nucleosome_model_0 (B, N)	2	2	2	1	1
C	fold_bknl2_sumo_model_0 (A,B)	1	0	1	0	1
D	fold_two_bknl2_two_sumo1_and_aknl2_model_0 (A, C)	2	1	2	0	2
E	fold_two_bknl2_two_sumo1_and_aknl2_model_0 (B, D)	2	2	2	0	2
F	fold_two_bknl2_two_sumo_model_0 (A, C)	2	0	2	0	3
G	fold_two_bknl2_two_sumo_model_0 (B, D)	2	0	2	0	3
H	fold_two_bknl2_one_sumo1_and_aknl2_model_0 (A, C)	2	1	2	0	4
I	fold_one_bknl2_one_sumo1_and_aknl2_model_0 (A, B)	1	1	1	0	5

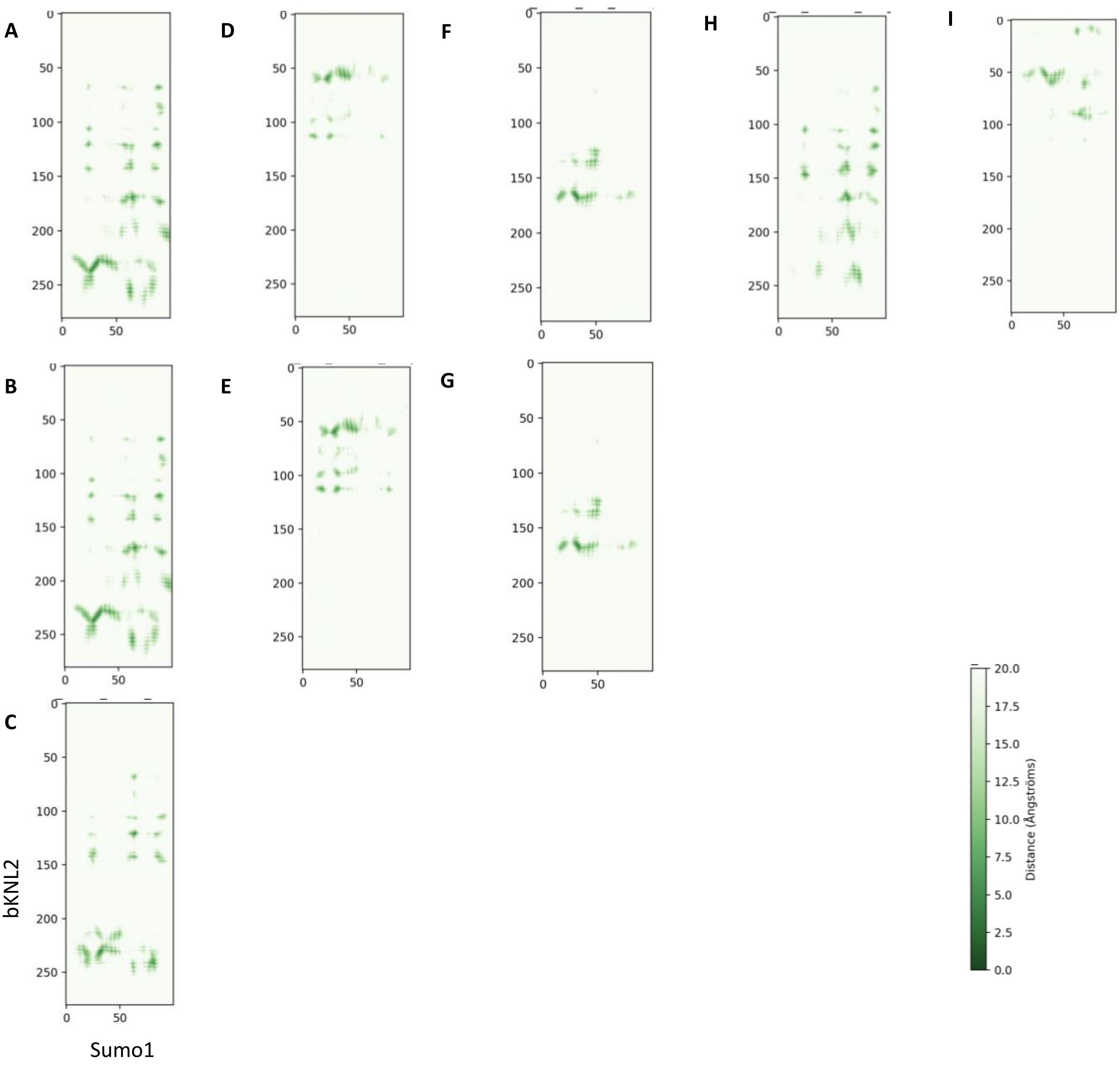

Supplementary Figure S15: Structural predictions with βKNL2 and SUMO1
The table lists all predictions where βKNL2 and SUMO1 are present, discriminating which and how many molecules. For each pair of βKNL2 and SUMO1 in the models there is one contact map below. βKNL2 residues are always in the y-axis and SUMO1 residues in the x-axis

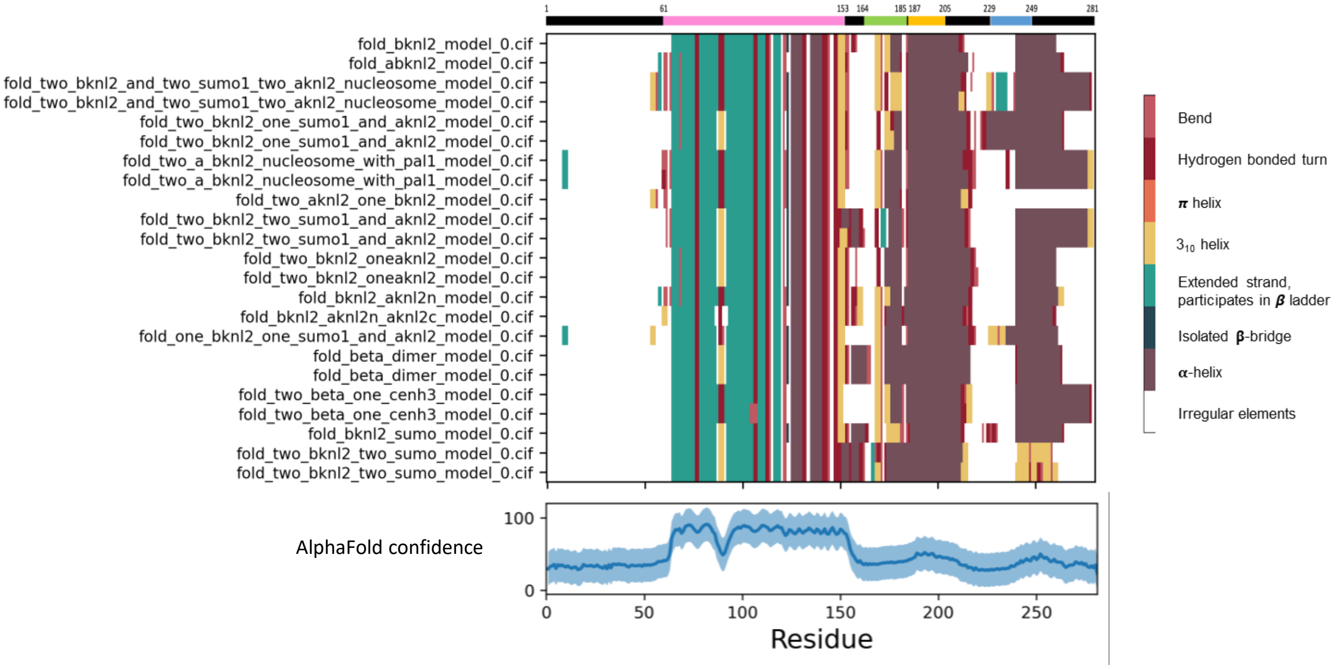

Supplementary Figure S16: Secondary structure and confidence of βKNL2 structural predictions
Secondary structure assignment to 23 molecules of βKNL2, present in the models listed in Sup. Fig. Models1, calculated with DSSP (ref) and colored according to the color bar on the right. At the bottom, the average (dark blue line) and standard deviation (light blue region) of AlphaFold's confidence calculated for each residue of the same models. On the top, the corresponding regions of the Santa domain (pink) and motifs I (green), II (yellow) and III (blue).

A

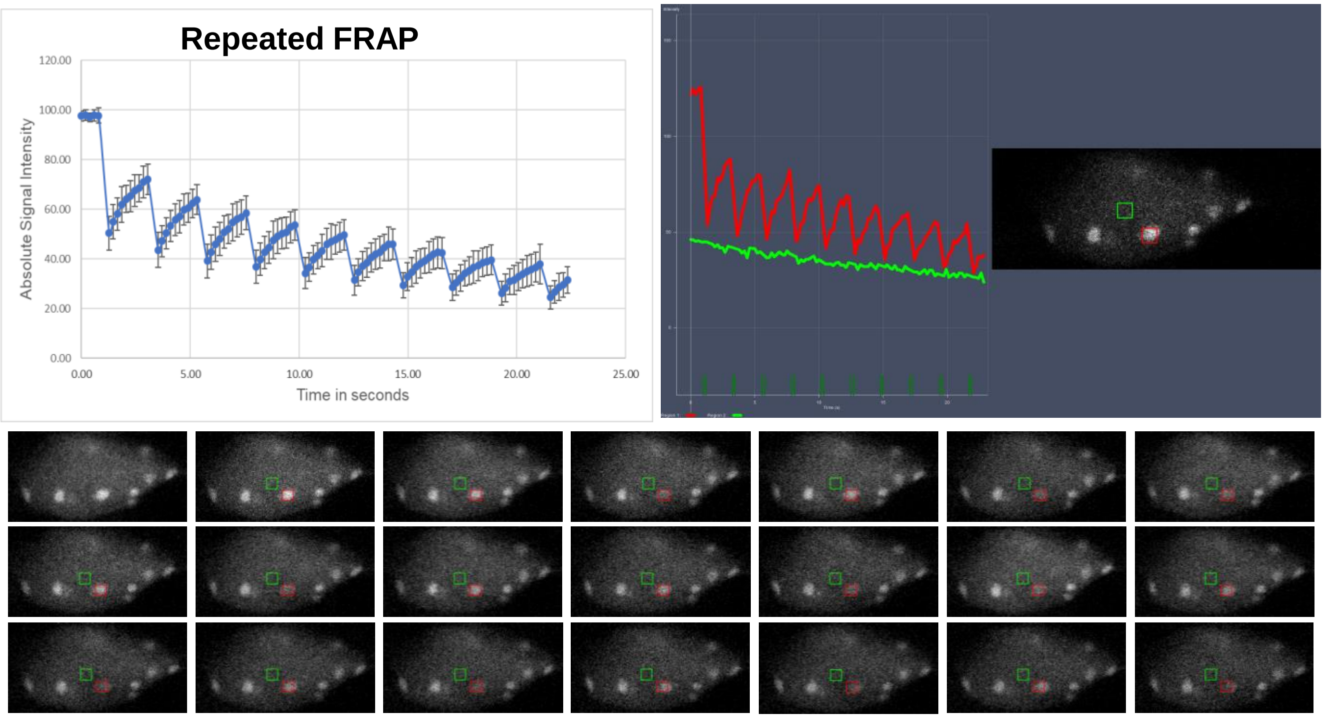

B

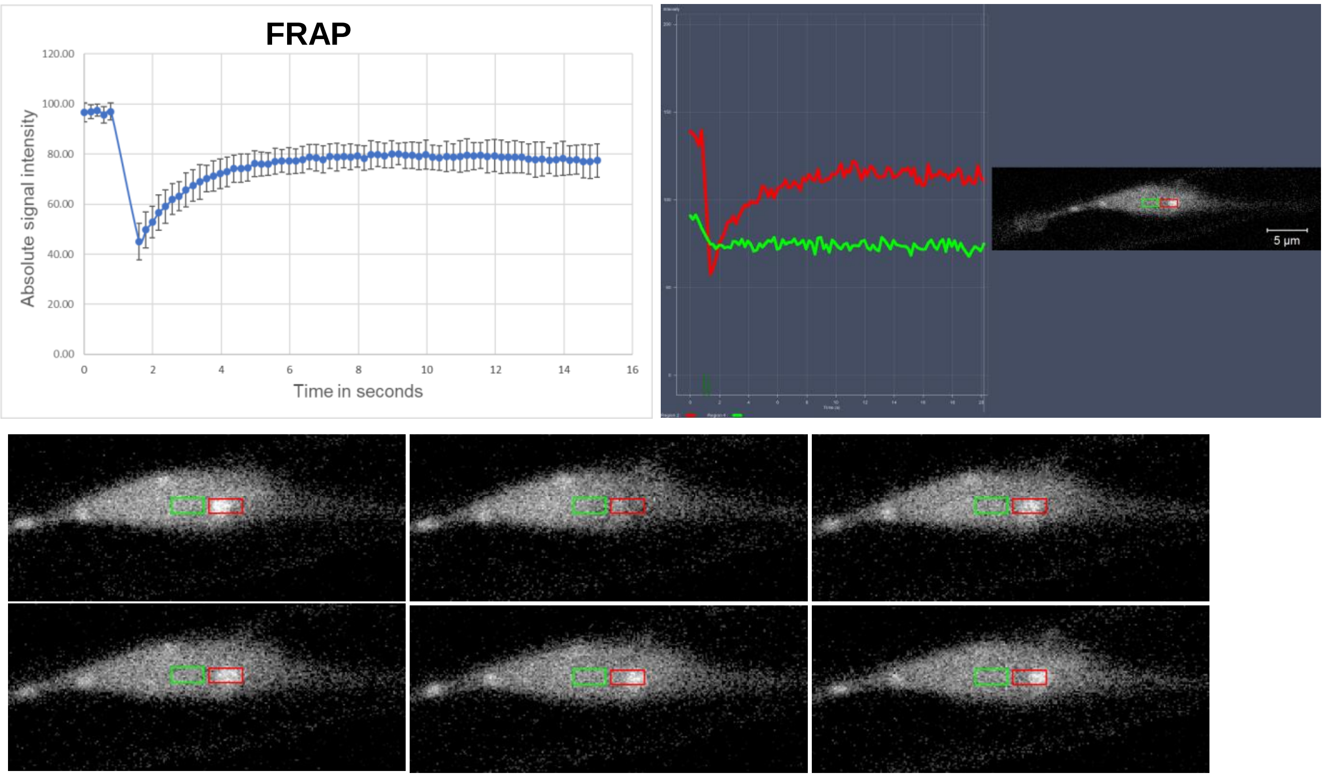

Supplementary Figure 17: FRAP experiments unveil the dynamic nature of βKNL2-EYFP. FRAP experiments were conducted in elongated non-meristematic nuclei of *A. thaliana* transformed with a 35S::βKNL2-EYFP construct. (A) For measuring repeated FRAP, individual nuclei were scanned three times with a 488 nm laser line (2,5% laser power, scan speed 6 without averaging) followed by repeated bleaching of a region of interest within the nucleus measuring 1,4 µm^2, using 100% laser power and 4 iterations alternated by single recordings. (B) For measuring FRAP, individual nuclei were first scanned three times with a 488 nm laser line (2.5% laser power, scan speed 6 without averaging). Following, a region of interest measuring 1.4 µm^2 was bleached by more than 50% and followed by 50 additional scans. Each experiment was run over the time scale of 40 sec, repeated 20–25 times and results averaged. In case of βKNL2 fluorescence intensity at chromocenters recovers to about 80% within 10 sec. For both FRAP experiments fluorescence intensity was measured in the area of bleaching (Bleach) as well as in adjacent regions. The x axis represents the time scale of the experiment in second, the y axis corresponds to fluorescence intensity (arbitrary units).

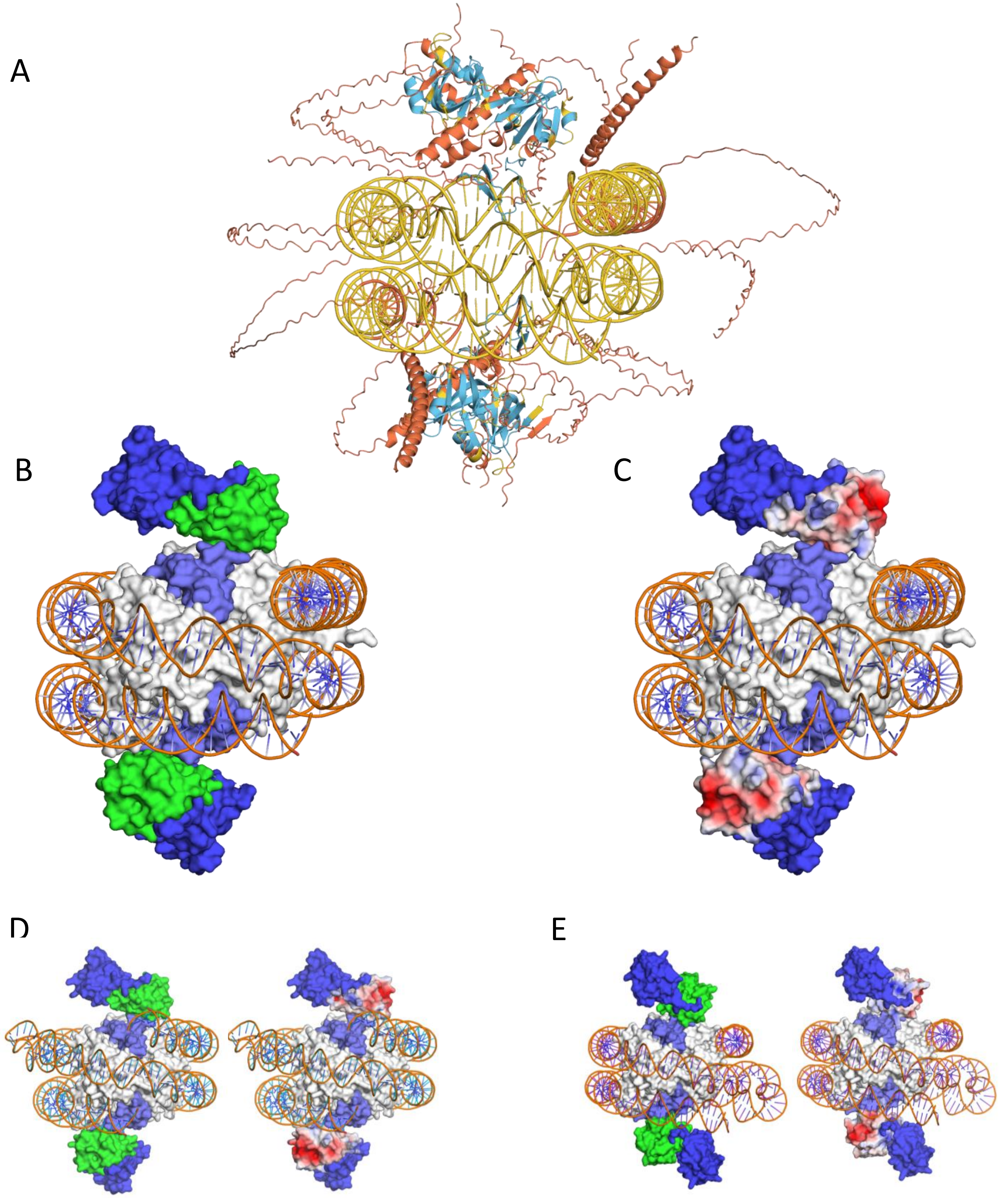

Supplementary Figure S18: Structural prediction of βKNL2 with αKNL2 and the nucleosome. (A) AlphaFold's prediction of two molecules of βKNL2 in complex with two molecules of αKNL2 and one nucleosome (fold_two_a_ βKNL2_nucleosome_histones_with_dissordered_reagions_model_0.cif) colored by AlphaFold's confidence. (B) Protein molecules in surface mode for only the regions with confidence higher than 50. These regions are the SANTA domain of βKNL2 (green), SANTA (dark blue) and CENPC-k (light blue) domains of αKNL2 and globular regions of all histones (white). (C) Same regions in surface mode, but βKNL2 is colored by surface charge distribution, from red (negative) to blue (positive). (D) Model fold_two_a_bknl2_nucleosome_with_pal1_model_0.cif and (E) Model fold_two_bknl2_and_two_sumo1_two_aknl2_nucleosome_model_0.cif aligned to the histones in (B) and (C), in the same representations.
