# Supplementary material for: Structural basis of βKNL2 centromeric targeting mechanism and its role in plant-specific kinetochore assembly
Source: Nucleic Acids Res. 2026 Jun 25;54(12):gkag605. doi: 10.1093/nar/gkag605 (PMC13294675; doi:10.1093/nar/gkag605)
Supplement: gkag605_Supplemental_Files [file gkag605_supplemental_files.zip › Supplementary tables.docx]

| Primers used for site directed mutagenesis | |
| --- | --- |
| βKNL2ΔN-For | ATGGTCACATTATCCGATTGGTGGCTAAC |
| βKNL2ΔN-Rev | GAAGCCTGCTTTTTTGTACAAAGTTGGC |
| βKNL2ΔSANTA-For | AATGAAGAAGAAGAAGAGAAGAAGAAGAAGAATGTTG |
| βKNL2ΔSANTA-Rev | GGATTTTAGGGTTTTGATTGGAGTGATGACAG |
| βKNL2ΔC-For | GACCCAGCTTTCTTGTACAAAGTTGGC |
| βKNL2ΔC-Rev | GTAATCTTCCCAATCATAAGGAAACCCTAAACG |
| βKNL2(C)-For | ATGAATGAAGAAGAAGAAGAGAAGAAGAAG |
| βKNL2ΔMotif I-For | TGTTTGAAAGATAAGATTTTGGACGATG |
| βKNL2ΔMotif I-Rev | CTTCTTCTTCTTCTCTTCTTCTTCTTC |
| βKNL2ΔMotif II-For | AAATCTGATAAGGCATGTGAGAAATCAAG |
| βKNL2ΔMotif II-Rev | ACAACCCTCAAGAGAATAAAGATCCTG |
| βKNL2ΔMotif III-For | TATGAAGCTTCTATTGGGAAAAGAGTTG |
| βKNL2ΔMotif III-Rev | ATCATCATCATCATCATCATCATCATCAAC |
| βKNL2ΔMotif III new-Rev | AACTCTTTTCCCAATAGAAGCTTCATAC |
| attB1+βKNL2 promote-756 | GGGGACAAGTTTGTACAAAAAAGCAGGCTTCGAGAGATCGGAAGCAAACGAC |
| attB2+βKNL2 promote-756 | GGGGACCACTTTGTACAAGAAAGCTGGGTCCCAACCGAAACTTCTTCTCCTA |
| Primers used for EMSA | |
| KNL2pf3aF | GGTTGCGATCGCATGGATTACAAGGATGACGATGACAAGGCAGCCGGTATGACGACGACGAGGGCGAAGTCCAA |
| KNL2pf3aR | GTGTGTTTAAACTTACCAACCGAAACTTCTTC |
| pAL1_f | GGTTAGTGTTTTGGAGTCGAATATG |
| pAL1_r | TTGCTTCTCAAAGATTTCATGGT |
|  |  |
| Primers used for genotyping and sequencing of constructs, colonies and transformants | |
| attB1 | GGGGACAAGTTTGTACAAAAAAGCAGGCT |
| attB2 | GGGGACCACTTTGTACAAGAAAGCTGGGT |
|  |  |
| Primers used for BIFC vectors construction | |
| BamHI 35S-For | ATGGATCCGTAAAACGACGGCCAGTGCCTAGC |
| MCS-35S-Rev | CTCGCATATCTCATTAAAGCAGTCTAGAACTAGTGAATTCGCGAAAGCTCGAGAGAGATAG |
| MCS-tocs-For | CTATCTCTCTCGAGCTTTCGCGAATTCACTAGTTCTAGACTGCTTTAATGAGATATGCGAG |
| PstI-tocs-Rev | TACTGCAGCTGCTGAGCCTCGACATGTTGTCG |
| Eco-VenN-For | TAGAATTCATGGTGAGCAAGGGCGAGGAGC |
| Spe-VenN-cmyc-Rev | TAACTAGTAAGATCCTCCTCAGAAATCAACTTTTGCTCCTCGATGTTGTGGCGGATC |
| Eco-VenC-For | TAGAATTCATGGACAAGCAGAAGAACGGCA |
| Spe-VenC-HA-Rev | TAACTAGTAGCGTAATCTGGAACATCGTATGGGTACTTGTACAGCTCGTCCATGCCGAGA |
| SpeI-cmyc-VenN-For | TAACTAGTATGGAGCAAAAGTTGATTTCTGAGGAGGATCTTATGGTGAGCAAGGGCGAGG |
| VenN-XbaI-Rev | TATCTAGACTACTCGATGTTGTGGCGGATCTTG |
| SpeI-HA-VenC-For | TAACTAGTATGTACCCATACGATGTTCCAGATTACGCTGACAAGCAGAAGAACGGCATC |
| VenC-XbaI-Rev | TATCTAGATTACTTGTACAGCTCGTCCATGCCG |
| SpeI-attR1-For | ATACTAGTTCAACAAGTTTGTACAAAAAAGCTG |
| SpeI-attR2-Rev | ATACTAGTAACCACTTTGTACAAGAAAGCTGAA |
| Primers used for *invitro* bacterial expression- SUMO assay | |
| βKNL FL (WT and Δmotif-III) to pET-Duet-FLAG-For | ACCATCATCACCACAGCCAGATGACGACGACGAGGGCG |
| βKNL FL (WT and Δmotif-III) to pET-Duet-FLAG-Rev | CTGAAAATACAGGTTTTCCGGCCAACCGAAACTTCTTCTCC |
| NSE2 to pET28 c+ For | GGTGGACAGCAAATGGGTCGGATCCCCATGGCGTCGGCGTCCTCG |
| NSE2 to pET28 c+ Rev | GGTGGTGGTGGTGGTGCTCGAGCTAATCTTCATCCACATCTTCTGTGAA |
| Primers used for Y2H assay | |
| aKNL2 1-118aa to pGBKT7-For | ATCTCAGAGGAGGACCTGCATATGatgacggaaccaaatctc |
| aKNL2 1-118aa to pGBKT7-Rev | GCGGCCGCTGCAGGTCGACGGATCCTTAttcaaagcaactgttacaaac |
| bKNL2 50-154aa to pGADT7-For | GACGTACCAGATTACGCTCATATGatgacaccgtttcccttag |
| bKNL2 50-154aa to pGADT7-Rev | GCAGCTCGAGCTCGATGGATCCTTAattgtaatcttcccaatcataag |

**Supplementary Table 1: List of all primers used in the study**

**Supplementary Table 2: List of all AlphaFold3 predicted models**

| **#** | **Model (chains of βKNL2 and αKNL2)** | **βKNL2** | **αKNL2** | **Sumo1** | **nucleosome** |
| --- | --- | --- | --- | --- | --- |
| A | fold_abknl2_model_0.cif (A, B) | 1 | 1 | 0 | 0 |
| B | fold_two_bknl2_and_two_sumo1_two_aknl2_nucleosome_model_0.cif (A, E) | 2 | 2 | 2 | 1 |
| C | fold_two_bknl2_one_sumo1_and_aknl2_model_0.cif (A, D) | 2 | 1 | 1 | 0 |
| D | fold_two_a_bknl2_nucleosome_with_pal1_model_0.cif (A, E) | 2 | 2 | 0 | 1 |
| E | fold_two_aknl2_one_bknl2_model_0.cif (A, B) | 1 | 2 | 0 | 0 |
| F | fold_two_bknl2_two_sumo1_and_aknl2_model_0.cif (A, E) | 2 | 2 | 2 | 0 |
| G | fold_two_bknl2_oneaknl2_model_0.cif (A, C) | 2 | 1 | 0 | 0 |
| H | fold_bknl2_aknl2n_model_0.cif (A, B) | 1 | 1 | 0 | 0 |
| I | fold_bknl2_aknl2n_aknl2c_model_0.cif (A, B) | 1 | 1 | 0 | 0 |
| J | fold_one_bknl2_one_sumo1_and_aknl2_model_0.cif (A, C) | 1 | 1 | 1 | 0 |
| K | fold_beta_dimer_model_0.cif (A, B) | 2 | 0 | 0 | 0 |
| L | fold_two_beta_one_cenh3_model_0.cif (A, B) | 2 | 0 | 0 | (1 CENH3) |
| M | fold_bknl2_sumo_model_0 (A,B) | 1 | 0 | 1 | 0 |
| N | fold_two_bknl2_two_sumo_model_0 (A, C) | 2 | 0 | 2 | 0 |
| O | fold_two_a_bknl2_nucleosome_with_dissordered_reagions_model_0.cif | 2 | 2 | 0 | 1 |
