# Supplementary figures and images for: Structural basis of βKNL2 centromeric targeting mechanism and its role in plant-specific kinetochore assembly
Source: Nucleic Acids Res. 2026 Jun 25;54(12):gkag605. doi: 10.1093/nar/gkag605 (PMC13294675; doi:10.1093/nar/gkag605)

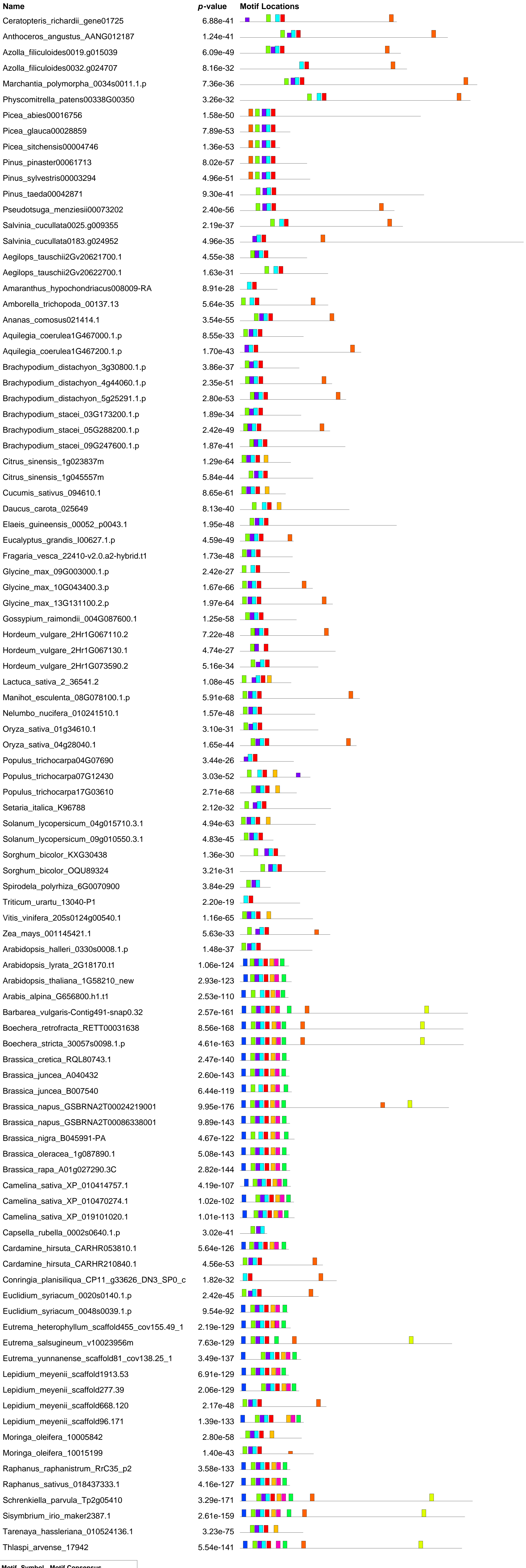

Supplement: gkag605_Supplemental_Files [file gkag605_supplemental_files.zip › Supp_File_2 motif_locations MEME.pdf]
